# Bioactive and Structural Metabolites of *Pseudomonas* and *Burkholderia* Species Causal Agents of Cultivated Mushrooms Diseases^1^

**Published:** 2008-05-09

**Authors:** Anna Andolfi, Alessio Cimmino, Pietro Lo Cantore, Nicola Sante Iacobellis, Antonio Evidente

**Affiliations:** 1 Dipartimento di Scienze del Suolo, della Pianta, dell’Ambiente e delle Produzioni Animali, Università di Napoli Federico II, Via Università 100, 80055 Portici, Italy; 2 Dipartimento di Biologia, Difesa e Biotecnologie Agro-Forestali, Università degli Studi della Basilicata, Viale dell’Ateneo Lucano 10, 85100 Potenza, Italy

**Keywords:** cultivated mushrooms and bacterial diseases, Pseudomonas tolaasii, P. reactans and Burkholderia gladioli pv. agaricicola, lipodepsipetides, mycopathogenic bacteria, antimicrobial activity, permeabilising effects on membranes, exopolysaccharides and lipopolysaccharides

## Abstract

*Pseudomonas tolaasii*, *P. reactans* and *Burkholderia gladioli* pv. *agaricicola*, are responsible of diseases on some species of cultivated mushrooms. The main bioactive metabolites produced by both *Pseudomonas* strains are the lipodepsipeptides (LDPs) tolaasin I and II and the so called White Line Inducing Principle (WLIP), respectively, LDPs which have been extensively studied for their role in the disease process and for their biological properties. In particular, their antimicrobial activity and the alteration of biological and model membranes (red blood cell and liposomes) was established. In the case of tolaasin I interaction with membranes was also related to the tridimensional structure in solution as determined by NMR combined with molecular dynamic calculation techniques. Recently, five news minor tolaasins, tolaasins A–E, were isolated from the culture filtrates of *P. tolaasii* and their chemical structure was determined by extensive use of NMR and MS spectroscopy. Furthermore, their antimicrobial activity was evaluated on target micro-organisms (fungi—including the cultivated mushrooms *Agaricus bisporus*, *Lentinus edodes*, and *Pleurotus* spp.—chromista, yeast and bacteria). The Gram positive bacteria resulted the most sensible and a significant structure-activity relationships was apparent. The isolation and structure determination of bioactive metabolites produced by *B. gladioli* pv. *agaricicola* are still in progress but preliminary results indicate their peptide nature. Furthermore, the exopolysaccharide (EPS) from the culture filtrates of *B. gladioli* pv. *agaricicola,* as well as the O-chain and lipid A, from the lipopolysaccharide (LPS) of the three bacteria, were isolated and the structures determined.

## Introduction

Brown blotch of *Agaricus bisporus* (Lange) Imbach (Tolaas, 1915) and the yellowing of *Pleurotus ostreatus* (Jacq.: Fr.) Kum (Ferri, 1985, pp 398; Iacobellis and Lo Cantore, 1997; Iacobellis and Lo Cantore, 1998a) are important diseases of the above cultivated mushrooms known to be caused by *Pseudomonas tolaasii* (Paine, 1919). However, recent studies have shown that the above diseases are complex ones. In particular, besides *P. tolaasii* also *P. reactans,* a saprotroph associated with cultivated mushrooms not yet classified and known in bibliography for its use in the white line assay for the specific identification of *P. tolaasii* (Wong and Preece, 1979), is responsible, though in different manner, of the symptom development on the hosts. Furthermore, *P. reactans*, has been shown to be the casual agent of the yellowing of *P. eryngii* (D.C.: Fr.) Quél a mushroom species cultivated in southern Italy (Iacobellis and Lo Cantore, 1997; Iacobellis and Lo Cantore, 1998b; Lo Cantore, 2001; Lo Cantore and Iacobellis, 2002; Iacobellis and Lo Cantore, 2003). The pathogenicity of *P. reactans* may be considered a novelty though indications in this regard have been reported in independent studies (Kim et al. 1995; Wells et al. 1996).

*Burkholderia gladioli* pv. *agaricicola* is the causal agent of the soft rot disease of the edible mushroom *A. bitorquis* on which it cause the rapid development of deep oozing lesions on the pileus which renders the mushroom unmarketable (Atkey et al. 1991, p 431; Lincoln et al. 1991). The disease appear geographically limited since it has been reported first in England (Lincoln et al. 1991) and then in New Zealand (Gill and Cole, 1992) on *A. bitorquis*. Preliminary analysis of the partially purified antimicrobial metabolites produced by of *B. gladioli* pv. *agaricicola* indicate their peptide nature. After that, the disease was reported on several common Japanese cultivated mushroom (Gill and Tsuneda, 1997).

## Isolation of Tolaasin I from Culture Filtrate of *Pseudomonas tolaasii*

**Figure 1:**
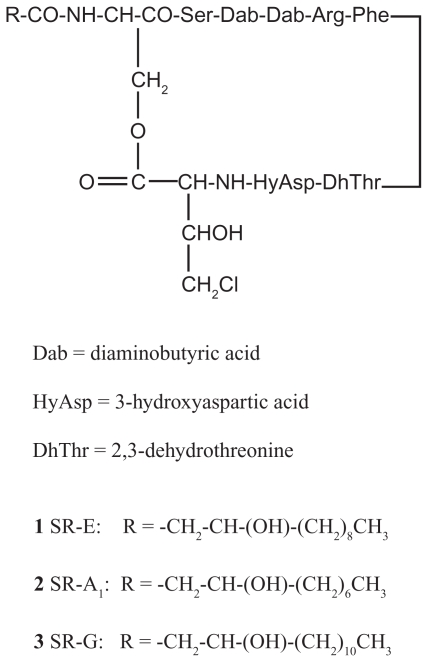
Structure of syringomicins A_1,_ E and G (**1**, **2** and **3**, respectively).

**Figure 2:**
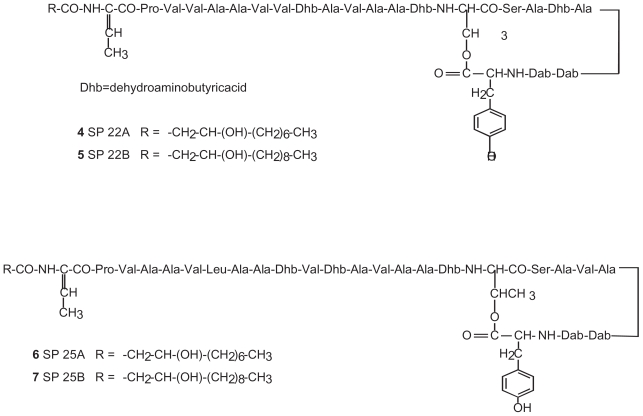
Structure of syringopeptins 22A, 22B, 25A and 25B (**4**, **5**, **6** and **7**, respectively).

Virulent strains of phytopathogenic as well as mycopathogenic *Pseudomonas* spp. produce *in vitro* toxic and antimicrobial lipodepsipeptides (LDPs) containing unusual aminoacids also with a d-stereochemistry and are classified in two groups according to their primary structures. The first group includes nonapeptides such as syringomycins (Fig. 1), syringotoxins, syringostatins and pseudomycins. The second group comprises molecules containing 18 to 25 amino acid residues, most of which having a d-stereochemistry, such as syringopeptins (Fig. 2), tolaasins, fuscopeptins and corpeptins, (Bender et al. 1999). In the latter group the C-terminal region forms a lactone ring of 5 (tolaasins, fuscopeptins and corpeptins) to 8 (syringopeptins) amino acids.

**Figure 3:**
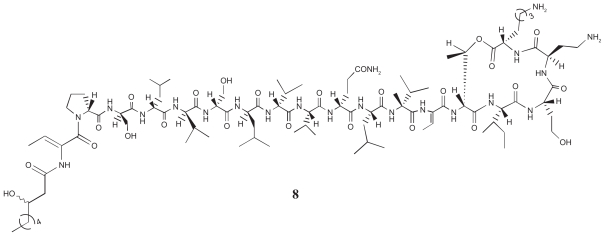
Tolaasin I (**8**) primary structure.

Tolaasin I (**8**, Fig. 3), a lipodepsipeptide produced in culture by virulent strains of *P. tolaasii* (Rainey et al. 1991), bears a β-OH octanoic acid blocking group at the N-terminus, a sequence of seven successive d-amino acids in the N-terminal region of the peptide (Pro 2 -Val 8), with a Ser-Leu-Val repeat, and then alternate l- and d-amino acids (Nutkins et al. 1991) (Fig. 3). It also contains a *Z*-dehydroalanine But) amino acid at positions 1 and 13, and a d-homoserine (Hse 16) and a d-2,4-diaminobutyric acid (d-Dab 17). Finally, a lactone ring is formed between the hydroxyl of d-Thr 14 and the C-terminal l-Lys 18.

Biological studies demonstrated that tolaasin I exhibits antimicrobial activity, strong resistance to enzymatic degradation and inactivation as well antigenicity (Rainey et al. 1991). This suggests for tolaasin I a potential role as a therapeutic peptide.

**Figure 4:**
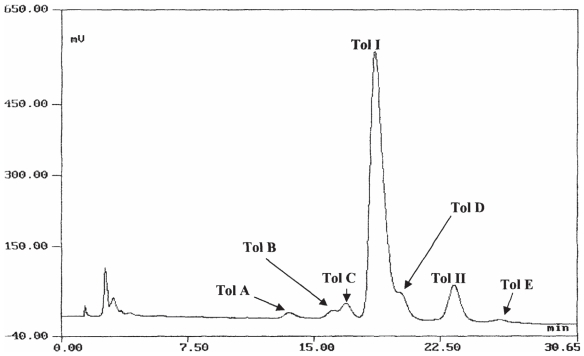
HPLC profile of crude tolaasins preparation.

Tolaasin I is considered the main virulence factor of *P. tolaasii* and responsible for the symptoms development on the mushrooms (Rainey et al. 1991; 1992; Soler-Rivas et al. 1999a) and this is apparently due to the ability of this molecule to disrupt cell membrane function by the transmembrane pore formation (Brodey et al. 1991; Rainey et al. 1991; Hutchinson and Johnstone, 1993). Tolaasin I was isolated from the cell-free bacterial culture filtrate of the type strain NCPPB2192 of *P. tolaasii* following a modification of the method described by Peng (1996). The further purification of the tolaasin I preparation by HPLC showed that *P. tolaasii,* besides the already known tolaasin I and II, which differ for the presence of Hse and Gly, respectively, in position 16, (Nutkins et al. 1991), produced *in vitro* other chemical correlated metabolites confirming previous findings (Shirata et al. 1995). HPLC profile of a crude tolaasin preparation (Fig. 4) carried out on an Analytical Brownlee Aquapore RP-300 (C8) column using water (0.1% TFA, v/v) and CH_3_CN as solvent system with a flow rates of 1.0 ml/min^−1^ [a Brown-lee Aquapore RP-300 (C8) column using the same solvent system with flow rate 4.7 ml/min was used for semipreparative purpose] showed the presence of five minor peaks, named tolaasins A, B, C, D, and E (Bassarello et al. 2004).

The chemical nature of tolaasin I was ascertained by comparing its NMR and MS data with those reported in the literature (Nutkins et al. 1991).

## Isolation of White Line Inducing Principle (WLIP) from Culture Filtrate of *Pseudomonas reactans*

**Figure 5:**
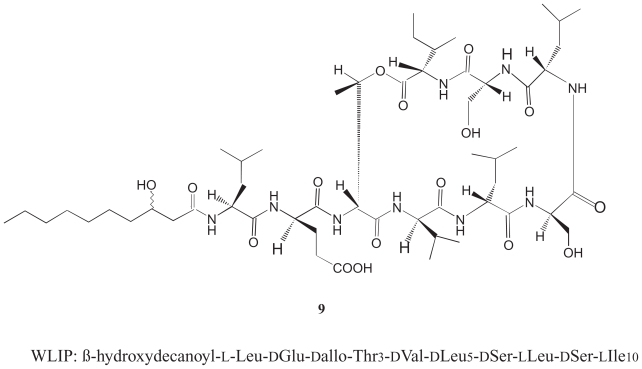
WLIP Primary structure (**9**).

Virulent strains of *P. reactans* produce in culture an extracellular substance called the White Line Inducing Principle (WLIP, **9**, Fig. 5), known for its ability to interact with tolaasin I and form a white precipitates in the “white line” assay useful for the identification of *P. tolaasii* (Wong and Preece, 1979). The ability of WLIP to precipitate tolaasin I, the main virulence factor of *P. tolaasii,* has been suggested to be useful for the control of brown blotches caused by *P. tolaasii* (Soler-Rivas et al. 1999b). WLIP, whose biological features are substantially unknown, is a lipodepsipeptide with a molecular weight of 1125 Da composed of a N-terminal β-hydroxydecanoic acid and a peptite moiety of nine aminoacids, six of which of d-form. The molecule presents a lactone ring between d-allo-threonine (d-Thr3) and N-terminal l-isoleucine (Ileu9) (Mortischire-Smith et al. 1991b). WLIP is structurally similar to viscosin (Laycock et al. 1991), except for the chirality of leucine in position 5 that it is d- in WLIP and l- in viscosin. This substance is a potent biosurfactant that appears important in the biology of the phytopathogenic bacterium *P. fluorescens* (Neu et al. 1990; Laycock et al. 1991).

The isolation and purification of crude WLIP from the culture filtrates of strain NCPPB1311 of *P. reactans* was performed according to the procedure reported by Mortishire-Smith et al. (1991b). Crude WLIP was obtained by fractionated precipitation in acid conditions whereas crystalline WLIP was prepared by the diffusion of water vapour into a solution of crude WLIP in methanol.

The identity of WLIP was ascertained by comparing NMR and MS data with those reported in literature (Mortishire-Smith et al. 1991b). The determination of the absolute structure of the lipodepsipetide by X-ray analysis allowed to ruled out its supposed identity with viscosin from which differ in the form of the crystalline cell.

Strain NCPPB1311 of *P. reactans* produced in culture high levels of WLIP with a yield of 169 mg/l which was more than ten fold higher of tolaasin I (13 mg/l) produced by strain NCPPB2192 of *P. tolaasii* (Lo Cantore et al. 2006).

## Biological Charaterization of Tolaasin I and of WLIP

### Antimicrobial activity of bacterial cultures and pure toxins

The antimicrobial assays of HCLP grade tolaasin I and crystalline WLIP confirmed the antimicrobial activity of tolaasin I (Rainey et al. 1991; 1992) and showed that also WLIP is a lipodepsipeptide inhibiting the growth of bacteria and fungi (Tables 1 and 2) (Lo Cantore et al. 2006). In general tolaasin I showed an antimicrobial activity higher than WLIP. The Gram positive bacterium *Bacillus megaterium* resulted the most sensible micro-organism to both tolaasin I and WLIP with a Minimal Inhibitory Quantity (M.I.Q) of 0.04 μg and 0.32 μg, respectively (Table 1). Also the other Gram positive bacteria used in this study were inhibited by both LPDs with M.I.Q significantly different on *Rhodococcus fascians* and *Curtobactrium flaccufaciens* (Table 1). On the contrary the Gram negative bacteria, including *Escherichia coli*, were inhibited by tolaasin I but not by WLIP (Table 1). It is of interest the fact that either cultures of strains NCPPB2192 or NCPPB1311 of *P. tolaasii* and *P. reactans*, respectively, inhibited the growth of *E. coli* K12 but only when the assays were performed on a minimal medium agar (MMA) (Lavermicocca et al. 1997). In fact, the above activity was not observed when the assays were performed on KB plates. The fact that the presence of the *E. coli* growth inhibiting metabolites was observed only on a minimal medium and the activity was reversed when peptone was added in the assay media suggest that either *P. tolaasii* and *P. reactans* produce besides tolaasins or WLIP, other metabolites, whose chemical nature and role in the biology of the producers should be investigated. It is not excluded that also the above bacteria produce metabolites similar to those produced by *P. syringae* strains involved in the inhibition of amino acids biosynthetic pathways (Bender et al. 1999; Iacobellis and Latorraca, 1999; Arrebola et al. 2003; Cazorla et al. 2003).

In general, filamentous fungi, including *A. bisporus, P. ostreatus, P. eryngii* and *Lentinus edodes,* were inhibited by both LPDs but on average tolaasin I was about eight/ten fold more active than WLIP (Table 2).

Furthermore, tolaasin I inhibited the growth of *Candida albicans* and *Cryptococcus neoformans*, yeast-like fungi responsible of systemic and cutaneous mycoses in animals and humans but the sensibility was lower when compared to the ones shown by phytopathogenic fungi. On the contrary, in the assay condition no activity on the growth of *C. parapsilosis* (Ashford) or *Malassezia pachydermatis* was observed (Lo Cantore et al. 2006).

The results of this study confirmed previous findings on the toxicity of tolaasin I on fungi (Rainey et al. 1991; 1992) and clearly demonstrated that also WLIP, as other LPDs of bacterial origin (Bender et al. 1999), is toxic on fungi though in general its activity is lower than that showed by tolaasin I. The above findings strongly suggest that also WLIP, as already reported for tolaasin I (Brodey et al. 1991; Rainey et al. 1991; 1992; Hutchison and Johnstone, 1993), is important in the microbial interaction with mushrooms. In this respect, when considering the observed lower antifungal activity of WLIP it is also necessary to remind that strain NCPPB1311 of *P. reactans*, at least *in vitro*, produced a quantity of WLIP which is more than ten fold higher the quantity of tolaasin I produced by the type strain NCPPB2192 of *P. tolaasii*. The lower antifungal activity of WLIP appear to be compensated by the higher quantity produced in culture. Furthermore, the observation that morphological variants of *P. reactans* strains are avirulent and do not produce WLIP (Lo Cantore, 2001; Iacobellis and Lo Cantore, 2003) further support the possibility that the latter compound may play an important role in the interaction of *P. reactans* with the cultivated mushrooms (Lo Cantore et al. 2006).

### Assay on tissue blocks and whole sporophores of *Agaricus bisporus*

**Figure 6:**
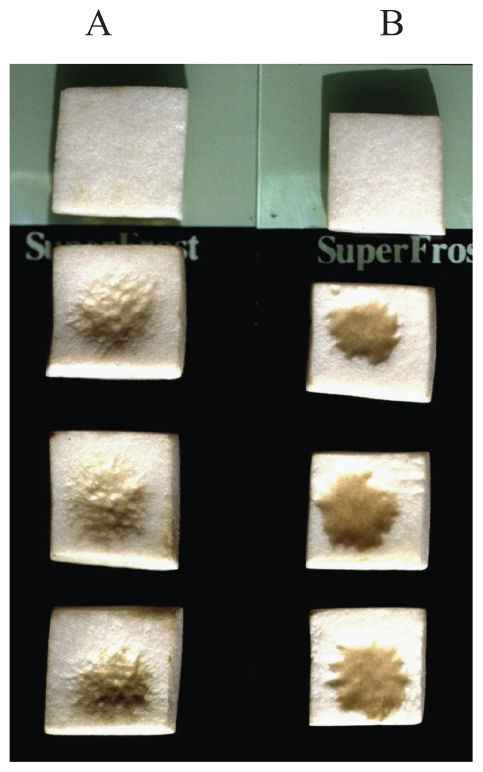
Brown lesions on tissue blocks of *Agaricus bisporus* (lower three blocks in each treatment), caused by deposition of 5 μl solutions containing 5.12 μg of WLIP (A) and 0.64 μg of tolaasin I (B), respectively. On upper blocks, 5 μl of sterile water was deposited.

Assays of the two LPDs on tissue blocks of *A. bisporus* showed that the deposition on their surface of a drop of solution containing 0.08 μg of tolaasin I caused brown sunken lesions. In the same assay condition a similar effect was observed when drops of solution containing 1.28 μg of WLIP were used (Fig. 6). Similar results were obtained when such LPDs have been deposited on the surface of whole sporophores caps (Lo Cantore et al. 2006).

### Haemolytic activity and osmotic protection

Agarose haemolysis assay showed that both WLIP and tolaasin I cause the red blood cells (RBCs) lysis (Table 3). however, the minimal haemolytic quantity of tolaasin I, in the assay conditions, was more than four fold higher than that of WLIP and, moreover than, as previously reported (Rainey et al. 1991), it was temperature dependent. However, this effect was not observed in the case of WLIP. The higher temperatures might facilitate the formation of oligomers of tolaasin I leading to the transmembrane pore formation (Lo Cantore et al. 2006).

Assays in water solution confirmed the higher haemolytic activity of WLIP. In fact, the minimal haemolytic concentration was 12.6 and 2.8 μM for tolaasin I and WLIP, respectively (Lo Cantore et al. 2006). In similar assays, Rainey et al. (1991) reported that 0.25 μM solution of tolaasin I determined an haemolytic effect. However, the latter experiments were performed on horse erythrocytes at 37 °C and also with the purpose to measure the lysis rate.

**Figure 7:**
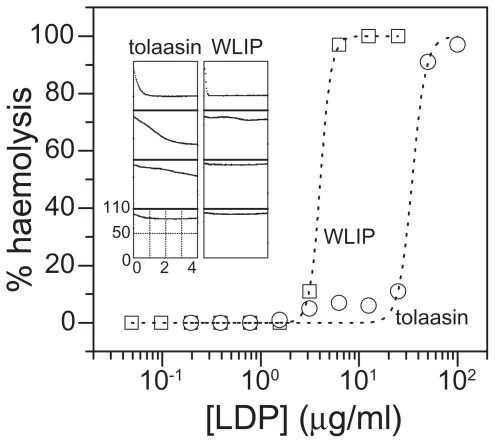
Dose dependence of the haemolytic activity of tolaasin I (circles) and WLIP (squares) after 4 hours at room temperature. Dotted lines are best fit experimental data points with Hill equation. Hill coefficients are 6.3 and 8.0 for tolaasin I and WLIP, respectively. **Inset**: Set of traces recorded on the microplate reader of kinetics of haemolysis of RBC exposed to 2 step sequential dilutions of tolaasin I (left) and WLIP (right), from top to bottom. Starting concentration (top panels) were 100 μg/ml for tolaasin I and 6.25 μg/ml for WLIP. The decrease of turbidity over time indicated the disappearance of intact RBC. All panels have the same linear axis, ranging from 0 to 4 hours and from 0 to 110 mOD for x- and y-axes, respectively.

In the solution assay, as shown in Figure 7, both tolaasin I and WLIP caused human RBCs lysis. WLIP was more effective than tolaasin I with regard to both C_50_ (i.e. the minimal concentration able to cause 50% haemolysis, which was close to 34.6 and 4.3 μg/ml for tolaasin I and WLIP, respectively) and of the haemolysis rate (Fig. 7, inset). The Hill coefficient associated to the formation of the active unit, appeared to be higher for WLIP (8 ± 2) than for tolaasin I (6 ± 1).

**Figure 8:**
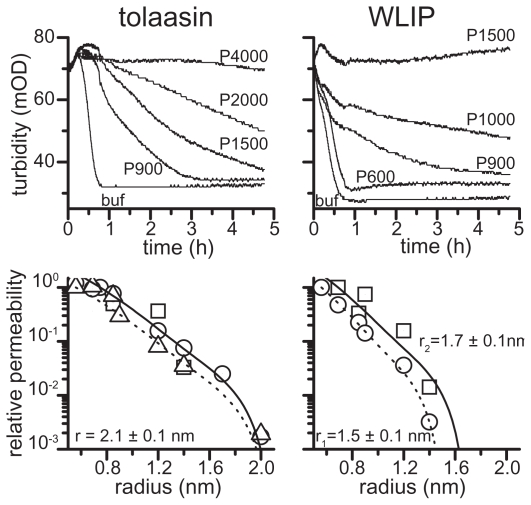
Haemolysis in the presence of osmotic protectants and Renkin fit. ***Upper panels***: Representative traces of decrease in turbidity of a RBC suspension in the presence of different external PEG osmolites and constant LDP concentrations (i.e. 100 μg/ml and 6.25 μg/ml for tolaasin I and WLIP, respectively). PEG size is indicated next to each trace. ***Lower panels***: Renkin representation of data collected in experiments similar to those reported in the upper panels. Curves through points are best fits according to Renkin equation [see text; Schultz and Solomon (1961); Ginsburg and Stein (1987)], which gives a pore radius for tolaasin I of 2.1 ± 0.1 nm. This value does not dependent on LDP concentration, at least at the concentrations used in this study (i.e. 50–100 μg/ml): dotted is the best fit of data collected at [tolaasin I] = 76 μg/ml (triangles, 2 × C_50_), solid line refers to best fit of data collected at [tolaasin I] = 50 (squares, 1.5 × C_50_) and 100 μg/ml (circles, 3 × C_50_). On the other hand, WLIP shows different values of pore radius, ranging from 1.5 to 1.7 nm depending on toxin concentration. Dotted line is the best fit of data collected at [WLIP] = 6.25 μg/ml (circles, 1.5 × C_50_), solid line refers to best fit of data collected at [WLIP] = 9 μg/ml (squares, 2 × C_50_).

The haemolytic activity of both LDPs can be prevented by adding osmoticants of adequate dimensions to the external medium. The results reported in Figure 8 suggest for WLIP and tolaasin I, at a concentration corresponding to 1.5 × C_50_, a pore radius between 1.5 ± 0.1 and 1.7 ± 0.1, and 2.1 ± 0.1 nm, respectively.

The small molecular size of the above LDPs suggests that such large pores could be formed by an aggregate of monomers, which is also consistent with the large Hill coefficients observed. The structure of the pore formed by WLIP appeared to be less stable with a more pronounced dependence on toxin concentration. In fact, at higher toxin dose (2 × C_50_) a bigger osmoticant is necessary for fully protect of RBCs from lysis (Fig. 8, right bottom panel) (Caraiola et al. 2006).

Theoretical analysis of the protection experiments with the Renkin equation permits an estimation of the functional pore radius which was 1.1 ± 0.1 and 2.0 ± 0.4 nm, for WLIP and tolaasin I, respectively. These radius values are similar to those found for other LDPs (Dalla Serra et al. 1999a). The previous smaller estimation of the functional pore radius of tolaasin I, reported by Rainey et al. (1991) to be 0.9 ÷ 1.0 nm, could depend on the lower tolaasin I concentration used in the experiments by the authors.

## The Solution Structure of Tolaasin I

### Secondary structure of tolaasin I

**Figure 9:**
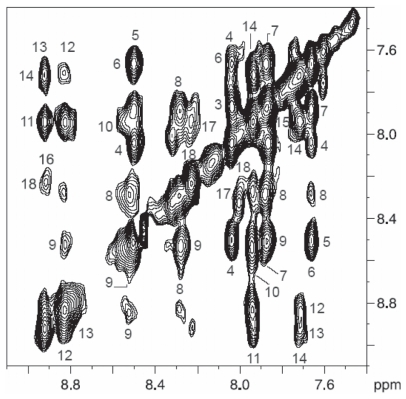
The amide region of NOESY spectrum of tolaasin I in ^1^H_2_O/^2^H_2_O (90/10 v/v) in the presence of SDS with a mixing time of 0.10 s, 300 K, pH 7.0. Where space permits, the cross-peaks are labeled with the sequence number of amino acid residues.

NMR spectra of tolaasin I were obtained in different solvents (^1^H_2_O, C^2^H_3_O^1^H, DMSO-d6, and aqueous SDS). Assignment of proton spin systems in all solvents used was obtained with the sequential methodology (Wüthrich, 1986). Tolaasin I secondary structure was delineated from the qualitative pattern recognition approach of the sequential and medium-range NOEs as derived from 2D experiments. In ^1^H_2_O and C^2^H_3_O^1^H, NOESY spectra essentially displayed sequential effects indicating that the conformation of the whole peptide was largely extended. In DMSO-d6, proton chemical shifts were found to correspond to those previously reported (Nutkins et al. 1991). NOESY spectra with 0.10 and 0.25 second mixing times showed some short- and medium-range NOEs in the region DSer3-DLeu7 in correspondence of tight turns as well as helical structure (Wagner et al. 1986). Most likely it was detected what is referred to as “nascent helix” (Dyson et al. 1988). The rest of the peptide proved to be essentially extended. The solution structure of tolaasin I was then studied in a ^1^H_2_O/^2^H_2_O (90/10 v/v) solution in presence of SDS at pH 7.0, a value at which the activity on erythrocytes is greatest (Raney et al. 1991). The amide region of the NOESY spectrum at 0.10 s mixing time is shown in Figure 9.

Sequential strong, non ambiguous cross-peaks between all consecutive amino acids, with the only exception of the dGln10-lLeu11, lIle15-lHse16, lHse16-dDab17 cross-peaks due to peak overlap were observed. Furthermore, several cross-peaks, namely dLeu4-dSer6, dVal8-dGln10, lLeu11-ΔBut13, dVal12-d*allo*Thr14 and lHse16-lLys18, were clearly observed. The presence of strong sequential NOEs together with medium in the region dPro2-lVal9 is a first indication of a helical structure. This NOE pattern, generally observed for right-handed helical structures in peptides and proteins with l-aminoacids, suggests here the presence of a left-handed helix.

Observation of several medium-range effects suggests the presence of an ordered structure. However, it is important to underline that the standard distances observed for helices, β-sheets and turns in peptides and proteins containing only d- or l-residues cannot be used here to infer simply the folding of tolaasin in the region containing alternate d- and l- amino acids. Not withstanding, the presence of key NOE effects between nonsequential residues of the same chirality hints at a turn like folding. In order to clarify the issue, the NOESY cross-peak intensities expected for a regular left-handed helix covering the 2–14 region, including the dPro2-dVal8 and the alternating l- and d-amino acids were calculated (Jourdan et al. 2003).

### Three-dimensional structure of tolaasin I

**Figure 10:**
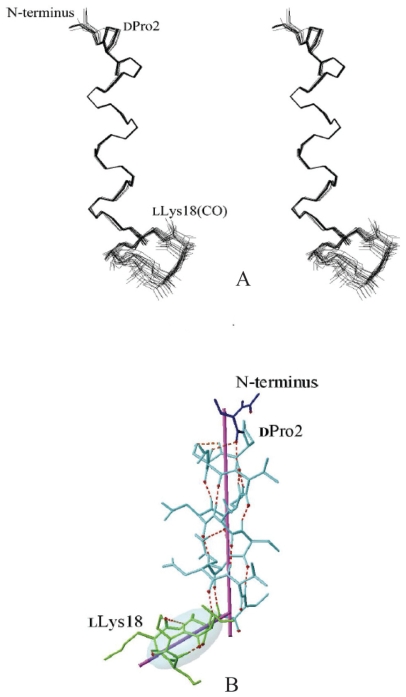
Calculated structures of tolaasin I (A) MOLMOL stereoview of the best-fit superposition of the 20 lowest energy rSA/rEMconformations of tolaasin. Backbone atoms of residues 2–14 were used for the best fit. (B) Stickgolf-club” like tolaasin I conformation. Dotted lines indicate hydrogen bondsalong the left-handed α-helix. The helical axis inside the helix is also represented. The shaded area simulates the ellipsoid that covers most of the backbone atoms of the lactone ring, and its largest axisis also shown.

In SDS micelles, tolaasin I forms an amphipathic left-handed α-helix in the region dPro2-dThr14. It comprises a sequence of seven d-amino acids (dPro2-dSer-dLeu-dVal5-dSer-dLeu-dVal8) and a stretch of l-d-l-d-d-amino acid residues (lVal9-dGln-lLeu-dVal-ΔBut-d*allo*Thr14) (Jourdan et al. 2003). A stereoview of the best fit superposition of the backbone atoms of the 20 rSA/rEM structures is shown in Figure 10A.

This is the first recognized example of a naturally occurring molecule with a regular left-handed α-helix including both d- and l-amino acids, and it could be relevant for the *de novo* design of protein structure. As a comparison, the closely related lipodepsipeptide syringopeptin 25-A also forms a left-handed α-helix, but only includes residues of d-chirality (Ballio et al. 1995). A percentage of 20% left-handed α-helix has been calculated from CD data, and it was hypothesized that the helix could be located in the N-terminal sequence of 7 d-amino acids (Mortishire-Smith et al. 1991a). However, the CD data do not allow to specifically position the helix in the sequence as 20% of the sequence represents only 3.6 residues, i.e. a single helix turn.

The C-terminal lactone loop adopts a “boat-like” conformation and is found shifted from the helical axis, therefore tolaasin I assumes a “golf-club” conformation (Fig. 10B) (Jourdan et al. 2003). This “golf club-like” overall conformation seems likely to anchor in plasma membrane by means of its amphipatic helical segment with the hydrophobic lactone loop lying close to the surface. Further studies remain highly desirable to understand how such a structure can create ion channels in plasma membranes. In particular, the formation of oligomeric structures, already hypothesized for syringomycin and syringopeptins, (Feigin et al. 1997; Hutchison and Johnstone, 1993; Dalla Serra et al. 1999a; 1999b) is now under investigation for tolaasin I.

It has been reported that the cationic heptapeptide ring in polymixyns (Storm et al. 1977), ranalexin (Clark et al. 1994), and in type-I brevinins (Simmaco et al. 1994) is responsible for the disruption of bacterial membrane permeability and thus for antibiotic activity. The octapeptide lactone ring of syringopeptins shows two positively charged residues as in the above molecules (Ballio et al. 1995). Also in the pentapeptidic C-terminal lactone ring in tolaasin I two positively charged groups (dDab17 and lLys18) are present. These findings imply that, independently from its size, the presence of positive charges into the loop together with the amphipathic helix, represent a fundamental membrane-permeabilizing motif (Oren and Shai, 1998; Andreu and Rivas, 1998).

### Structure-activity relationships of tolaasin I

It has been reported that tolaasin I is able to cause horse erythrocytes haemolysis as consequence of transmembrane pores formation in erythrocyte (Raney et al. 1991). Because of its small size this effect may be the result of the monomer aggregation across the membrane. Zn^2+^ inactivates ion channel formation probably via chelation of ionizable groups in the membrane near the site of the pore formation (Brodey et al. 1991). Tolaasin I secondary structure suggests that the amphipathic N-terminal α-helix is deeply anchored into the membrane, leaving the hydrophilic lactone ring close to the surface of membrane. A longer anchored tail might both result in a higher affinity to the membrane and a stronger disrupting effect. Moreover, the length of the N-terminal sequence might play a central role in the formation of stable oligomeric structures required for ion channel formation. This may explain the loss of activity in ion channels formation observed in the case of tolaasin 144, an altered tolaasin I derivative lacking dSer6-dLeu7-dVal8. Preliminary MD simulations indicate that the N-terminal sequence of tolaasin 144 is more flexible than tolaasin I and it does not fold into a stable α helix. These feature may prevent the oligomeric aggregate formation and may explain the fact that tolaasin I lyses erythrocytes by two mechanisms (Hutchison and Johnstone, 1993). At low tolaasin I concentration the activity is the result of Zn^2+^-sensitive ion channel formation whereas at high concentrations it is the result of Zn^2+^-insensitive detergent action of both molecules. It has been reported that the α-helical structure, while important for cytotoxicity toward mammalian cells, is not a prerequisite for antibacterial activity (Shai and Oren, 1996). Novel antibiotic peptides useful as therapeutic drugs require strong antibiotic activity against bacterial and fungal cells without haemolytic effect (Raney et al. 1991). Functional and structural studies with d-amino acids incorporated analogues of pardaxin reveal that the lack of a significant α-helix does not prevent the lysis of bacterial membrane. The peptide is probably acting as a detergent in what has been described as a “carpet”-like mechanism (Shai, 1995; Shai and Oren, 1996). Therefore, the decrease of the helical content into lytyc peptides by incorporating d-amino acids may be a useful approach to discover new peptide antibiotics for therapeutic use. In this context, tolaasin I and tolaasin 144 are very interesting molecules: the presence of a stable α-helix in the former warrants its lytyc activity, while the decreased helical content together with the detergent action exerted by tolaasin 144 strongly suggest its possible role as antibacterial, but not cytotoxic, peptide. Furthermore, the above substances present l-amino acids into a d-amino acid sequence: this induces resistance to enzymatic degradation and inactivation (Wade et al. 1990), and increase antigenicity (Benkirane et al. 1993). The antimicrobial assays of tolaasin I, though limited to a representative set of test micro-organisms, confirmed the high sensitivity of Gram-positive bacteria, as previously reported (Raney et al. 1991), and showed a quite good activity toward the yeast *Rhodotorula pilimanae* and the Gram negative bacterium *E. coli* (Lo Cantore et al. 2003a; 2006). The activity against *E. coli* (strain ITM100), in contrast with the results previously reported (Raney et al. 1991), indicates a variability of sensitivity among strains, and strongly suggests that before conclusions are drawn several strains of the same micro-organisms must be checked. It would be of interest to test tolaasin I and tolaasin 144 against Gram-positive and Gram-negative bacteria of medical interest. Finally, the structural information on tolaasin I can be extremely helpful for its possible use in the control of the brown blotch disease of *A. bisporus* also in combination with other methods. Treatment of mushrooms caps with tolaasin I provokes pitting and browning (Raney et al. 1991) as well as activation of the defense mechanism of the mushroom (Soler-Rivas et al. 1999a). Nutkins et al. (1991) reported that partially purified toxin reproduces disease symptoms, as does the intact organism, although tolaasin I induces more prominent symptoms than living bacteria (Moquet et al. 1996). Accordingly, tolaasin I appears to be an adequate target to stop the sequence of events affecting *A. bisporus*. The fact that WLIP appears to be able to inhibit the browning could confirm this hypothesis (Jourdan et al. 2003).

## Isolation and Chemical and Biological Chracterization of News Tolaasins

**Figure 11:**
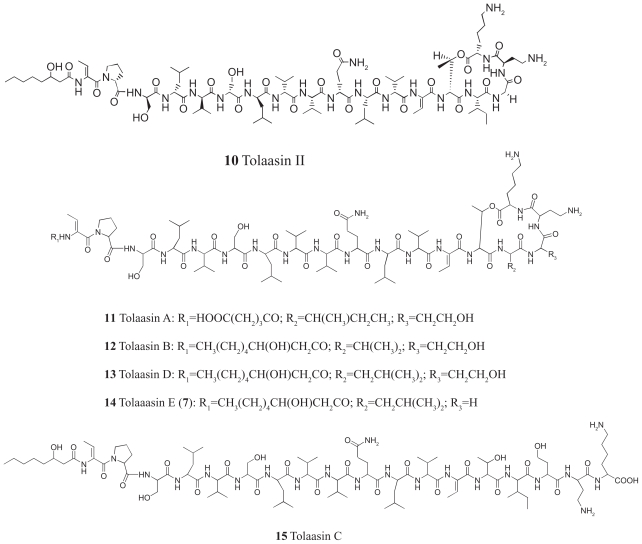
Structures of tolaasins II and A–E (**10**–**15**).

Tolaasins A–E (**11**–**15**, Fig. 11), were isolated together with tolaasins I and II from the cell-free culture filtrate of the type strain NCPPB2192 of *P. tolaasii* essentially by semi-preparative HPLC as above cited (Fig. 4).

A preliminary analysis of ^1^H NMR and ESIMS data of these metabolites indicated that the new lipodepsipetides are structurally related to tolaasins I (**8**) and II (**10**) as previously suggested by Shirata et al. (1995). The authors isolated eight tolaasins, including tolaasins I and II from *P. tolaasii* cultures filtrates and hypothesized, only on the basis of MS data, that their structural features were similar to **8** and **10**. In our case seven tolaasins, including tolaasins I and II, were isolated and the structures of two of them differ from those hypothesized from Shirata et al. (1995). A complete NMR-based structural elucidation of tolaasins A–E (**11**–**15**) was not an easy task to accomplish, due to the low amount of substances available. For this reason, while a full characterization of the ^1^H-NMR spin systems was achieved through extensive use of 2D NMR techniques, ^13^C NMR carbon chemical shifts could not be assigned. Attempts to aquire ^13^C NMR data by means of suitable 2D NMR experiments (HSQC and HMBC) at very long-acquisition time failed. The assignment of the proton spin systems of each amino acid residue, including the identification of the N-terminal side chain, was mainly obtained from the 2D TOCSY and the 2Q (Double quantum spectroscopy) experiments (Wüthrich, 1986; p 292). The 2Q spectrum was acquired in order to assess the sequence specific assignment of the spin systems preliminarily characterized in the TOCSY spectrum, together with the pattern analysis of sequential dipolar couplings displayed in the 2D NOESY spectrum (Wüthrich, 1986; p-292). Full proton assignments for tolaasins A–E (**11**–**15**), in DMSO-d6 at 300 K were obtained (Bassarello et al. 2004).

**Tolaasin A** (**11**) showed a pseudomolecular ion peak in the HRESIMS spectrum at *m/z* 980.0912 (M+2H)^2+^, consistent with the molecular formula C_91_H_155_N_21_O_26_ (exact mass calculated for C_91_H_157_N_21_O_26_/2 980.080435). HRESIMS data were obtained also for the intense fragment ion (M-ϕchain-ΔBut1+H) and interpreted as originating from the elimination of [HOOC-(CH_2_)_3_CONHC=CH(CH_2_)-C=O] with hydrogen transfer to the charged species (see below). Extensive analysis of the ^1^H NMR data of tolaasin A (**11**), including TOCSY, DQF COSY, and 2Q spectra, permitted confirmation of the same amino acid sequence present in the parent tolaasin I (**8**). Moreover, the Thr14 residue and the C-terminal Lys18 were found to be involved in the lactone formation, as already observed for tolaasin I. On the other hand, a different nature of the N-terminus ϕ chain was suggested by the analysis of the DQF-COSY spectrum. The N-terminus blocking group was unequivocally identified by the fragmentation peaks observed in the ESIMS spectrum and appeared to have a HOOC-(CH_2_)_3_-CO- structure. Finally, the ESI MS/MS (collision induced dissociation) spectrum, confirmed the same amino acid sequence for the two depsipeptides.

**Tolaasin B** (**12**) showed a pseudomolecular ion peak at *m/z* 1973 (M+H)^+^, 14 Daltons less than tolaasin I, suggesting that tolaasin B could differ from the analog tolaasin I by the absence of a methylene group. NMR and the ESIMS data permitted to deduce for tolaasin B a molecular formula C_93_H_161_N_21_O_25_. Moreover, several peptide fragmentation peaks generated by a stepwise spontaneous loss of amino acid residues from the N-terminus were observed. The TOCSY spectrum, almost superimposible to that of tolaasin I, permitted the identification of tolaasin B (**12**), which shows the substitution of the isoleucine spin system present in tolaasin I (**8**) with a proton connectivity consistent with a valine residue. Two intense NOE correlations were observed between Hse16 NH and Val Hα, and Val NH and Thr14 Hα, confirming that the valine residue is at position 15. The presence of the intact lactone moiety was confirmed by CID MS/MS analysis. Taken together the above data allowed to establish the structure **12** for tolaasin B.

**Tolaasin C** (**15**) showed a pseudomolecular ion peak at *m/z* 2005 (M+H)^+^ corresponding to a molecular weight of 2003 Da, 18 mass units greater than that of tolaasin I, suggesting that tolaasin C may be the acyclic version of tolaasin I derived by the hydrolysis of its lactone ring (molecular formula C_94_H_165_N_21_O_26_). Comparison of 2D NMR spectra and the ESIMS fragmentation patterns of tolaasins C (**15**) and I (**8**) further supported the hypothesis that tolaasin C is a linear peptide generated from the hydrolysis of the lactone ring. In fact, the proton chemical shifts of the dehydroamino-butyric acid residue at position-13 (ΔBut13) and the threonine at position-14 suggested that the hydroxyl group of the side chain of this residue is not involved in an ester functionality.

**Tolaasin D** (**13**) exihibited a pseudomolecular ion peak at *m/z* 1987 (M+H)^+^, suggesting the same molecular formula C_94_H_163_N_21_O_25_ already observed for tolaasin I. Thus tolaasin D (**13**) and I (**8**) are isomers. A careful comparison of the TOCSY spectra of tolaasin D and I indicated that tolaasin D contained a leucine residue in place of the isoleucine present in the related lipodepsipeptide **8**. The 2Q spectrum allowed to specifically assign the resonances of the leucine methylene protons. The position 15 of the leucine was supported by two NOE effects between Hse16 NH and Leu15 Hα, and between Leu15 NH and Thr14 H α.

**Tolaasin E** (**14**) showed a pseudomolecular ion peak at *m/z* 1943 (M+H)^+^ suggesting the same molecular formula C_92_H_159_N_21_O_24_ of tolaasin II (**10**). Hence, tolaasin E (**14**) is isomeric with tolaasin II (**10**). The latter depsipeptide, contains a glycine residue in place of the homoserine residue of tolaasin I (**8**). In fact, in the TOCSY spectrum of tolaasin E it was possible to characterize the spin system of a glycine residue, confirming that **14** is indeed a tolaasin II analogue. The 2Q experiment indicated the presence of four rather than three leucine residues. A careful examination of the NOESY and TOCSY spectra confirmed that the first three leucine residues are at positions 4, 7 and 11, as in tolaasin II (**10**). NOE correlations allowed the placement of the fourth leucine at position 15 in tolaasin II. A support to the structure of tolaasin E was the presence of the β-hydroxyoctanoil ϕ chain at the N-terminus as deduced by ESIMS data through the spontaneous loss of 225 Da (the ϕ chain + ΔBut1) from the pseudomolecular ion peak at *m/z* 1943.

The antimicrobial activity of HPLC grade tolaasins A-E (**11**–**15**)—assayed in comparison with tolaasin I and II against the yeast *R. pilimanae*, the fungus *Rizoctonia solani*, the Gram-positive bacteria *B. megaterium* and *Rodococcus fascians*, respectively, and the Gram-negative bacteria *E. coli* and *Erwinia carotovora* subsp. *carotovora—*is reported in Table 4. *B. megaterium* and *R. fascians* were the most sensible test micro-organisms. In fact, all the analogs, except tolaasin C, inhibited the growth of these bacteria though differences among their specific activities were observed. The most active analogs appear to be tolaasin D followed by tolaasin I and II with a minimal inhibitory quantity of 0.16, 0.32 and 0.64 μg, respectively. Tolaasins A and B, and E were less active, with a minimal inhibitory quantity of 1.28 and 2.56 μg, respectively. A similar sensitivity to the above substances was shown by the fungus *R. solani.* None of the analogs inhibited the growth of the Gram-negative bacteria *E. coli* and *E. carotovora* subsp. *carotovora* at least at the concentration used in the assays. Finally, the growth of the yeast *R. pilimanae* was inhibited only by tolaasins I, II, A and D though the sensitivity of the yeast was lower in comparison to *B. megaterium* (Table 4). The results of the antimicrobial activity of the tolaasins suggest the importance in this respect of the lactone and the N-terminus acyl moiety. Tolaasin A, which has the pentadioic acid instead of the β-hydroxyoctanoic acid, showed a reduced activity in respect to tolaasin I while tolaasin C, the linear derivative of tolaasin I, due to the lactone opening, lacked the activity at least at the concentration tested. The effect of the lactone ring opening on the lack of the antimicrobial activity has been already reported for other bacterial lipodepsipeptides (Jourdan et al. 2003). Furthermore, the importance of the position 15 in the peptide moiety of both tolaasins I and its derivatives in respect to the antimicrobial activity was observed. In fact, the replacement of isoleucine in position 15 with a valine or leucine residue in tolaasins B and D, respectively, determines the decrease or the increase of the antimicrobial activity when compared to tolaasin I. The presence of leucine in position 15 in tolaasin E determined the reduction of the activity when compared to tolaasin II. Though the latter result is apparently in contrast with the effect of the same replacement in the tolaasin D, it is important to consider that tolaasin II differ from tolaasin I for the replacement of the homoserine in position 16 with a glycine residue (Bassarello et al. 2004).

As previously reported tolaasin I forms an amphipatic left-handed α-helix in the region dProd2-d*allo*Thr14 comprising the sequence of seven d-amino acids and the adjacent l-d-l-d-d-region. Furthermore, the lactone macrocycle adopts a “boat-like” conformation and is shifted from the helical axis giving rise to a “golf-club” overall conformation. These structural features appear of importance on toxicity of tolaasin I (Jourdan et al. 2003). Therefore, structural changes, occurring in position 15 in the case of tolaasins B and D, in position 16 in tolaasin II, and in both position 15 and 16 in tolaasin E, may modify this lactone conformation and consequently the antimicrobial activity (Bassarello et al. 2004).

## Mode of Action of Tolaasin I and WLIP on Natural and Model Membranes

The activity WLIP and tolaasin I was comparatively evaluated on lipid membranes. Both LDPs induced the release of calcein from large unilamellar vesicles (LUVs). Their activity was dependent on the toxin concentration and liposome composition and in particular it increased with the sphingomyelin (SM) content of the membrane. Studies of dynamic light scattering suggested a detergent-like activity for WLIP at high concentration (>27 μM). This effect was not detected for tolaasin I at the concentrations tested (<28 μM). Differences were also observed in LDPs secondary structure. In particular, the conformation of the smaller WLIP changed slightly when it passed from the buffer solution to the lipid environment. On the contrary, a valuable increment in the helical content of tolaasin I, which was inserted in the membrane core and oriented parallel to the lipid acyl chains, was observed. The novelty of the pathogenetic capability of *P. reactans* and, in particular, the fact that avirulent variants of the pathogen have also lost the ability to produce WLIP (Lo Cantore and Iacobellis, 2002) suggest that this metabolite may be important in the *P. reactans*-mushrooms interactions. Although the haemolytic and the antimicrobial activity of WLIP and its interactions with synthetic membranes have been recently demonstrated (Lo Cantore et al. 2003a, p 255–62; Lo Cantore et al. 2003b, p 263–73; Lo Cantore et al. 2006), the mechanism involved is not yet defined. The effects of WLIP on model membranes of different lipid composition, also in comparison to tolaasin I, were investigated analysing the LDPs mode of action and the molecularity of the per-meabilising unit. In addition, the changes in the secondary structure occurring when these molecules passed from the aqueous buffer to the membrane were studied. In particular, it was possible to analyse the conformation of WLIP and tolaasin I during their interaction with the lipid membrane, i.e. their natural target, and, in the case of tolaasin I, to calculate the molecule orientation with respect to the lipid acyl chains (Coraiola et al. 2006).

### Calcein release assay

Both WLIP and tolaasin I are able to damage biological and artificial membranes through the formation of transmembrane pores (Brodey et al. 1991; Cho and Kim, 2003; Lo Cantore et al. 2003b, p 263–73; Lo Cantore et al. 2006).

**Figure 12:**
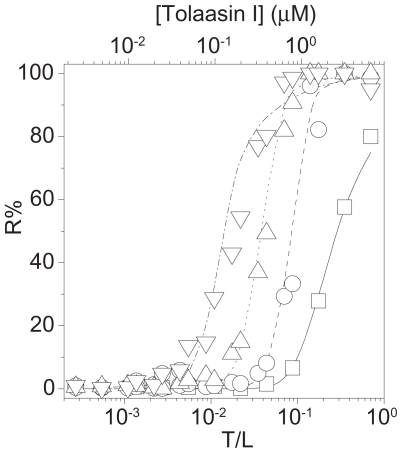
Dose dependence activity of tolaasin I on liposomes with different lipid composition: Calcein loaded liposomes were exposed to different peptide concentrations. The percentage of calcein release was determined after 45 min as a function of the toxin/lipid ratio (T/L) and expressed as % of the maximal value obtained with TritonX-100. Experiments were done at constant lipid concentration (about 9 μM). The true toxin concentration used was reported in the upper scale of the panel. Lines through the points are best fit according to the statistical model described in the text. Best values of fitting parameters are reported in Table 1. Vesicles were prepared with the following compositions expressed in molar ratio: (down triangle) PC:SM (50:50), (up triangle) PC:SM:Chol (50:33:16.5), (circle) PC:SM:Chol (50:16.5:33), (square) PC:Chol (50:50).

Tolaasin I was more active on SM- and phospholipids containing LUVs and less active when sterols were present (Table 5 and Fig. 12). This feature, observed for other peptin-like LDPs (Dalla Serra et al. 1999b; Menestrina, 2003, p 185–98), was correlated to their cell specificity being peptin-like LDPs more phytotoxic and less antifungal than the smaller LDPs such as the nonapeptides syringomycines, pseudomycines and cormycin. The latter showed a clear preference for sterols (Bender et al. 1999; Dalla Serra et al. 1999b; Menestrina 2003; Julmanop et al. 1993; Taguchi et al. 1994; Feigin et al. 1997; Scaloni et al. 2004). In the case of tolaasin I, the slight preference for ergosterol (a fungal sterol) among sterols is consistent with its antifungal activity.

On the contrary to the expectations, also WLIP, which is a nonapeptide, was more active on SM-containing LUVs. This could be explained by the fact that this LDP shows structure similarity with the peptine-like LDPs with only a portion of the peptide moiety involved in the formation of the lactone ring. In comparison to tolaasin I, the inhibitory effect of sterols was less effective on the WLIP activity.

Nevertheless the two toxins showed similar responses to changes of the lipid membrane composition.

Firstly, the molecularity of the permeabilising unit, estimated from the Parente-Rapaport model and reported in Table 5, was larger with WLIP and in the same range of detergents (Dalla Serra et al. 1999a). In the case of tolaasin I, Cho and Kim (2003) described the existence of subconductance states, which could be related to the presence of pores with slightly different molecularities. This instability of the pore structure well correlates with the variability of the parameter M found by our analysis (Coraiola et al. 2006).

For both of LDPs the activity seemed mainly due to the ability of monomers to bind to the liposome membrane (K_1_) rather than to the two-dimensional aggregation rate (K_2_) of the bound peptide (Caraiola et al. 2006).

### Dynamic light scattering

**Figure 13:**
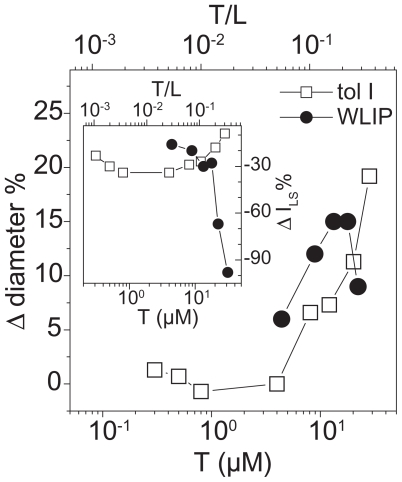
Effect of toxins on vesicle size as determined by dynamic light scattering: The treatment of phosphatidylcholine vesicle with tolaasin I (open square) and WLIP (close circle) caused changes in vesicle average size (Δ diameter) which was reported vs the toxin concentration (T), normalized to the vesicle diameter in the absence of toxin. Experiments were obtained at constant lipid concentration (80 μM). The toxin/lipid ratio (T/L) was reported in the upper scale of the same panel. **Inset.** In this case we analyzed the variation of the scattered light intensity (Δ I_LS_), normalized to the intensity value in the absence of toxin. Other parameters are as above.

From the results reported in Figure 13 it was evident that WLIP solution at concentration higher than 27 μM showed a detergent-like activity; in fact, the intensity of the scattered light lowered probably in consequence of vesicles micellization. On the contrary, both LDPs at low concentration (at a LDP to lipid molar ratio up to 0.3) caused an increase of the liposome dimensions and a decrease of the intensity of the diffused light. These effects well correlated with vesicles aggregation, that increased the measured diameters, and with precipitation of vesicle macro-aggregates which caused a decrease of signal intensity through a decrease of the concentration of scattering centres.

Interestingly, the concentrations of WLIP and tolaasin I used in these assays were of the same order of magnitude required to induce mushroom tissue alteration *in vivo* (Lo Cantore et al. 2006; Brodey et al. 1991; Hutchison and Johnstone, 1993). Also the different activity between the two LDPs was observed, being WLIP about ten times less active than tolaasin I (Lo Cantore et al. 2003a; 2006).

### Fourier-transformed infrared spectroscopy experiments

The results reported in above paragraphs together with those in Lo Cantore et al. (2006), supported the idea of the cell membrane as the main target for both WLIP and tolaasin I. Therefore it seemed of interest to investigate the conformational changes occurring when these molecules interact with the lipid environment. The secondary structure of both LDPs was already reported but only in the soluble form (Mortishire-Smith et al. 1991b; Jourdan et al. 2003). In particular, the conformation of tolaasin I was studied in SDS (Mortishire-Smith et al. 1991a, 1991b; Jourdan et al. 2003): in this membrane-like environment it was demonstrated the presence of a left-handed α-helix, suggesting a refolding of the structure from the aqueous buffer where the conformation of the whole peptide was largely extended (Jourdan et al. 2003). Considering these results, FTIR spectroscopy was used to determine the LPDs conformation and to follow its changes when the molecules passed from hydrophilic to a membrane mimetic environment. For this reason, FTIR spectra were collected in buffer solution, in an organic solvent (TFE, HFIP), in SDS or in the presence of lipid vescicles. The structure of peptides was analyzed in the lipid-mimetic conditions provided by TFE, HFIP and SDS, as well as in the model lipid membrane of palmitolyl-oleoyl-phosphatidylcholine (POPC) liposome. Changes in the secondary structure of peptides passing from the water buffer solution to the more hydrophobic environment with a maximum effect in the presence of the lipid membrane were expected (Table 6).

**Figure 14:**
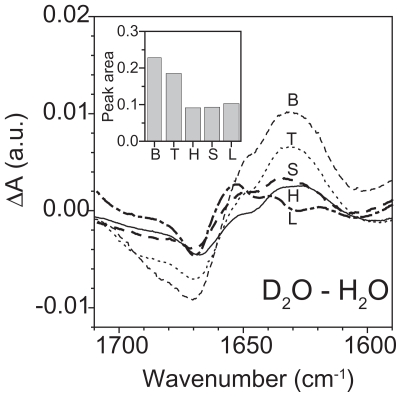
Differential spectra after H/D exchange of the WLIP amide protons: Analysis of differences in the amide I band of films of WLIP samples deposited from a buffer solution (B, dashed line), TFE (T, dotted line), HFIP (H, solid line), SDS (S, thick dashed line) and after binding to the lipid membrane (L, thick dotted-dashed line). Differential spectra were obtained by subtracting the hydrogenated spectra of WLIP in the different environments from the corresponding deuterated spectra. (Inset) Area of negative peaks between 1710 and 1660 cm^−1^. All the differences are between normalized spectra, i.e. with the amide I’ peak area set at 1.

**Figure 15:**
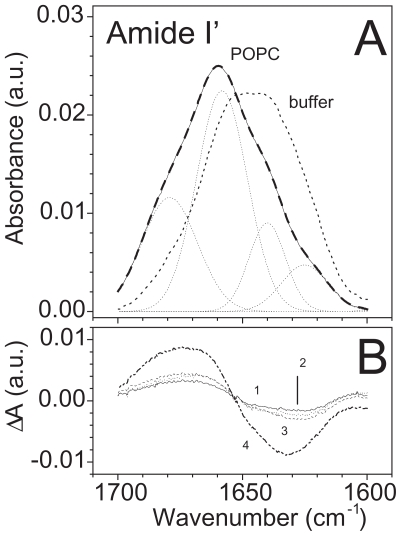
FTIR-ATR spectra of WLIP in buffer and in lipid mimetic environments: (A) Analysis of the amide I’ band of deuterated films of WLIP samples deposited from a buffer solution (buffer, thick dotted line) or after binding to the lipid membrane (POPC, solid line). The original spectrum (POPC) was deconvoluted and curve fitted to resolve the component frequencies. The corresponding Gaussian bands are reported as dotted lines, and their sum as a thick dashed line superimposed to the original spectrum. (B) Differential spectra were obtained by subtracting the deuterated spectrum of the soluble form in buffer from that in TFE (1), in HFIP (3), in SDS (2) or in presence of the lipid phase (4).

The rigidity of WLIP, cyclized via lactone formation between the third and the C-terminal residues (Mortishire-Smith et al. 1991b), could explain the slight variation observed in the amide I’ spectrum even after the interaction with the membrane. Moreover, the presence in the same molecule of unusual residues made the assignment of a secondary structure to the curve fitting components more difficult. Therefore the differential spectra obtained after H/D exchange or after the buffer substitution with a membrane-like environment were focused (Fig. 14). In fact, variations of spectra after H/D exchange provided information about the local structure of the molecule. The decrease of absorption at 1670 cm^−1^ (and the corresponding increment at 1634 cm^−1^), for example, indicated the presence of free protons (i.e. not involved in hydrogen bonds) which were able to exchange with deuterium, probably because of their good accessibility allowed by the local structure of the molecule. This behaviour could be attributed to a turn configuration with fast exchanging protons. Interestingly, this behaviour was dependent on the solvent used and decreased in a lipid-mimetic environment (effect measured by the small negative and positive peaks at 1670 cm^−1^ and 1634 cm^−1^, respectively), suggesting a decrease of the proton accessibility and, consequently, a change in the local structure of the molecule (Fig. 15A). Therefore, it seemed that the lipid-mimetic conditions enhanced the formation of hydrogen bonds inside the structure which become less susceptible to deuteration. Similar changes were confirmed in differential spectra obtained by subtracting the deuterated spectrum of the soluble form in buffer from those obtained with other solvents (Fig. 15B). Variations in the absorption at the above wavenumbers showed an increment at 1670 cm^−1^ and a corresponding decrease at 1634 cm^−1^ with the maximum in the lipid membrane. These changes favoured a more stable local structure which was enhanced from the lipid-mimetic environments and that could be able to interact with the membrane and its hydrophobic moiety. In addition, the disordering effect on the vesicles membrane induced by the WLIP binding supports the hypothesis of an insertion of the molecule into the membrane lipid core.

**Figure 16:**
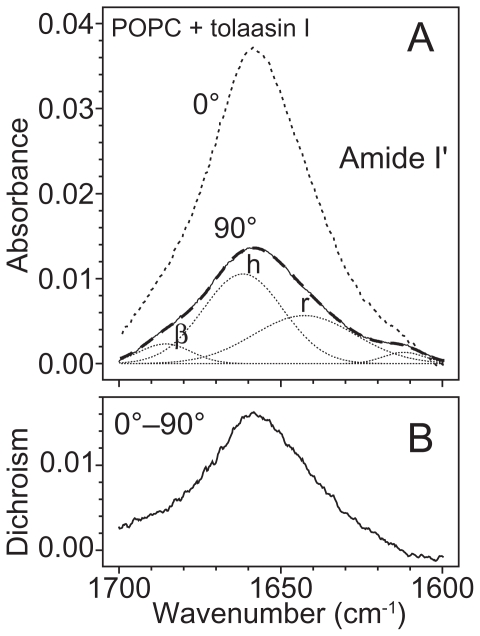
Analysis of FTIR-ATR spectra of tolaasin I in POPC layers with polarizer: (A) Spectra were taken with either parallel (0°) or perpendicular (90°) polarization. The amide I’ region of tolaasin I bound to vesicles was reported after subtraction of the lipid contribution. The best fit curve with Gaussian components (dotted lines) was superimposed as a thick dashed line to the 90° polarized trace (solid line). The absorption bands in the parallel and perpendicular configuration were used to calculate the orientation of the corresponding structural element as reported in Table 7. Bands are: h (helix), β (β-structure), r (random). (B) Dichroic spectrum obtained by subtracting the 90° polarized spectrum (after multiplication by R_iso_ i.e. 1.54) from that at 0°.

As expected an increment of the helical content in the structure of tolaasin I was observed when it passed from the water buffer solution to the membrane-like environment. This is consistent with results obtained by CD and NMR spectroscopy in aqueous solution and in SDS, respectively (Mortishire-Smith et al. 1991a; Jourdan et al. 2003). Nevertheless some considerations need to be added. The peptide moiety of tolaasin I (18 aminoacids) includes unusual residues with d-chirality, so the standard assignment of secondary structures to the curve fit components could be not always applied. Interesting recent studies were recently reported on α-helical membrane lytic peptides, in which a few l-amino acids were replaced with their d-enantiomers: incremented amide I’ frequencies (1656–1670 cm^−1^) compared with pure α-helical structures (1645–1654 cm^−1^) were observed and assigned to 3_10_-helix or dynamic/distorted α-helix (Papo and Shai, 2004). Previous studies had correlated this region with 3_10_-helix (Kennedy et al. 1991), β-turn (Susi and Byler, 1986) and interestingly to a left-handed α-helix, as well (Giancotti et al. 1973; Citra and Alxelsen, 1996). In this analysis two components around the typical region of helix absorption, centred at 1654 cm^−1^ and 1664 cm^−1^, respectively (data not shown) were found (Fig. 16A and Table 7). After deuteration the position of both peaks did not change, suggesting a stable structure maintained by hydrogen bonds, like a helix. Moreover, the sum of percentage absorption of these two components kept at a constant value, independently of the environment (i.e. buffer, solvents and membranes), on the contrary the single absorptions changed: the peak at 1654 cm^−1^ increased passing from buffer to solvents at expenses of peaks at 1664 cm^−1^. After interaction with the membrane, the contribution of the former was maximum but analysis of the dichroic spectrum showed only one peak at higher wavenumber (1659 cm^−1^) (Fig. 16B). For all these reasons, the two contributions were joined in an unique peak and assignto it a helix structure which finally resulted the conformation adopted from tolaasin I to bind the membrane and to insert in it. The significant decrease in the orientation order parameter of the aliphatic chain region (Table 6) and the orientation of tolaasin I in the lipid bilayer prompt to interpret these results in favour of a barrel-stave mechanism of action versus a carpet like hypothesis (Malev et al. 2002). This is indeed in agreement with Bassarello et al. (2004) who described for tolaasin I the formation of a stable amphipathic helical structure and suggested its insertion into the lipid matrix for the formation of an ionic channel.

On the contrary, a carpet-like mechanism was previously proposed for magainin derived lipopeptides which localized on the membrane surface and did not significantly destabilise the acyl-chain order. In fact, Avrahami and Shai (2002) observed negative order parmeters for both natural magainin and lipo-magainin (magainin analogues conjugated with lipophilic acids), typical of helices oriented nearly parallel to the membrane surface. In addition, after incorporation of the peptides into the lipid membrane, the lipid order was only poorly disturbed and to the same extent, suggesting that both peptides did not insert deeply into the lipid core.

A different mechanism of action could be proposed for WLIP, in agreement with the evidences here reported for membrane disruption in a detergent-like manner. In fact, it does not have a sufficient length to transverse the entire membrane, similarly to syringomicin for which Malev et al. (2002) hypothesized a toroidal pore.

In conclusion, the results described shed light on the permabilising effects induced by WLIP and tolaasin I on liposome and on the secondary structure of these molecules during their interaction with the lipid membrane, which is their biological target (Caraiola et al. 2006).

## Exo- and Lipo-Polysaccharide from Pseudomonas tolaasii, P. reactans and Burkholderia gladioli pv. agaricicola

In the last decades the chemical composition and structures of exopolysaccharides (EPSs), complex molecules of the bacterial capsules, of several plant pathogenic bacteria have been determined. Some EPSs have a very complex structure, being heterogeneous branched heteropolymers. The differences in the chemical composition and structures of the EPSs purified from different plant pathogens suggest that these molecules may play specific and different roles in plant-pathogen interaction (Evidente and Motta, 2002, p 581–629).

The involvement of extracellular polysaccharidic or glycopolysaccharidic substances in bacterial and fungal plant diseases has often been reported (Hodgson et al. 1949; Harborne, 1983, p 743–82; Denny, 1995; van Alfen, 1989) but their role in the disease process is still to be clarified (Denny, 1995; van Alfen, 1989).

EPSs are considered either molecules able to avoid or delay the activation of plant defence, acting as signal in plant pathogen cross-talk as well as they may have a significant role in the virulence of the pathogen of interest. In several phytopathogenic bacterial species belonging to the genus *Agrobacterium*, *Clavibacter*, *Erwinia*, *Pseudomonas* and *Xanthomonas*, the production of polysaccharidic or glycopolysaccharidic substances was proved to be associated with water soaking and/or wilting symptoms and, moreover, they played an important role in plant colonisation by the bacterium (Denny, 1995). Furthermore, EPSs from *X. campestris* pv. *vesicatoria* were proved to induce phytotoxic effects (chlorosis, necrosis, electrolyte leakage) on the homologous host (Walkes and Garro, 1996).

These macromolecules appear to interfer with water movement in plant due to mechanical plugging of the vessels which leads to wilt symptoms development. The phenomenon appears to be related to molecule size and viscosity rather than to their structure (Harborne, 1983, p 743–82), though some results on their apparent host specificity (Rudolph et al. 1989, p 177–218) and on viscosity interference (McWain and Gregory, 1972) would suggest a possible different behaviour in at least in some cases.

Lipopolysaccharides (LPSs) are complex molecules of the outer membrane of Gram negative bacteria containing a polysaccharide (hydrophilic) tail and a lipid (hydrophobic) head, which is anchored in the outer membrane. The molecule consists of three segments: lipid A, core polysaccharide, and the antigenic O-chain. The core polysaccharide is further subdivided into an “inner” core and an “outer” core. The three segments differ in their composition, biosynthesis and biological function. Although this model of LPS was originally proposed for *Salmonella* spp., the basic features appear valid for most, if not all, Gram-negative bacteria (Chatterjee and Vidaver, 1986, p 94–114).

The isolation and structure determination of LPSs from phytopathogenic bacteria, and in particular that of the corresponding O-chain, assumed great importance for the possible role of this molecule in the host-pathogen interaction. LPSs appear to play an important role in the interaction of cells of pathogenic bacteria and plant and animal cell hosts (Medzhiton and Janway, 1997, 1998.; Dow et al. 2000). LPSs activate host defence systems in either vertebrate and invertebrate inducing the production of antimicrobial peptides or, in mammalian, that of immunoregulatory, inflammatory and cytotoxic molecules. The use of mutants of plant pathogenic bacteria defective or lacking LPSs lead to assess their role in the virulence expression and in the recognition process which takes place in the first phases of the interaction of the pathogen and plant cells.

The O-chain and lipid A, from the LPS of *P. tolaasii*, *P. reactans* and *B. gladioli* pv. *agaricicola* as well as the EPS of the latter was isolated and the structures determined.

### Lipopolysaccharide of *Pseudomonas tolaasii*

**Figure 17:**
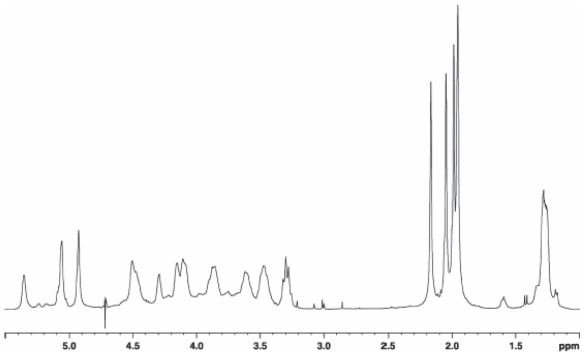
^1^H NMR spectrum of the O-chain of the LPS from *Pseudomonas tolaasii.*

Dried cells of type strain NCPPB2192 of *P. tolaasii* were extracted according to the water/phenol method (Westphal and Jann, 1965) and the LPS fraction was found exclusively in the phenol phase. The LPS material was subjected to vertical gel electrophoresis and it appeared as a simple ladder like pattern, typical of a regular repetitive lipopolysaccharide species of smooth LPS form. A mild acid hydrolysis allowed the removal of the lipid A moiety by precipitation leaving the polysaccharide in solution which was then purified by gel filtration. The compositional analysis of pure O-polysaccharide, via acetylated *O*-methyl glycoside derivatives, showed the presence of 2-acetamido-2-deoxy-gulu-ronic acid and 2-acetamido-2,6-di-deoxy-glucose (Qui*p*N). Methylation analysis showed the presence of 3-substituted quinovosamine. The ^1^H NMR spectrum of the polysaccharide showed in the anomeric region a variety of signals not all attributable to anomeric protons and not integrating for the same area (Fig. 17). Therefore, a detailed 2D NMR analysis (DQF-COSY, TOCSY, NOESY, gHSQC, gHMBC) allowed the complete assignment of all resonances. Interestingly, the HSQC spectrum showed the signals of some spin systems (**A** and **A’**) and (**B** and **B’**) and these were indicative of their anomeric positions.

**Figure 18:**
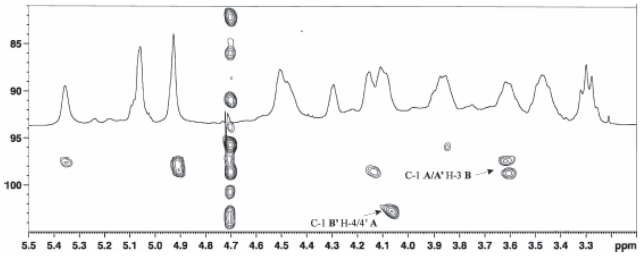
Relevant HMBC correlation of the anomeric region of the O-chain from *Pseudomonas tolaasii.*

A gHSQC spectrum registered without decoupling allowed the measurement of the ^1^*J*_C,H_ anomeric coupling, thus demonstrating that spin system **A** and **A’** have α-configuration whereas the spin system **B** and **B’** have β-configuration. Residue A had typical proton and carbon signals of a uronic acid substituted at C-4 whereas residue A’ had proton and carbon resonances of a 4-substituted 3-*O*-acetyl uronic acid. The value of the coupling constants and the ^13^C chemical shifts were distinctive of a *gulo*-configurated hexopyranose (De Castro et al. 2003). The other spin systems (**B** and **B’**), showing the same resonances and both characterised by the anomeric signals, were identified 3-substituted β-Qui*p*NAc. The acetyl substitution at C-2 of all residues and at C-3 of residue **A’** was proven by a HMBC spectrum. The sequence of the monosaccharides in the repeating unit was inferred using interresidual NOE data measured by 2D-NOESY and long range scalar connectivities measured by HMBC spectrum (Fig. 18).

Selective and milder de-O-acetylation conditions were carried out (ammonium hydroxide at 4 °C for 4 days) yielded a polysaccharide with a disaccharide repeating unit as demonstrated by 2D NMR analysis. This experiment demonstrated 2-acetamido-2-deoxy-gulopyranuronamide and 2-acetamido-2,6-di-deoxy-glucopyranose as constituents of the disaccharide unit. The absolute configuration of these residues was determined by using both NMR and the Exciton Coupling Method approaches (Harada and Nakanishi, 1983) carried out on the corresponding *p*-bromobenzoyl derivatives of O-methyl glycosides N-acetylated obtained by a strong methanolysis of O-chain followed by acetylation.

The absolute configuration of both gulopyra-nose units derivative was established on the basis of ^13^C chemical shifts of the de-acetylated derivative. The analysis has been carried out considering the two possible relative configurations for the compound in the disaccharide repeating unit, that is: →4)-α-L-Gul*p*-NAcAN-(1→3)-β-d-Qui*p*NAc-(1→ and →4)-α-d-Gul*p*NAcAN-(1→3)-β-d-Qui*p*NAc-(1→. The ^13^C chemical shifts of gulopyranose residue univocally indicate L-configuration in agreement with previous data (Lipkind et al. 1988; Shashkov et al. 1988).

Hence, the O-chain of the LPS from the mushrooms associated bacterium *P. tolaasii* consists in a tetrasaccharide repeating unit built up of two units 2-acetamido-2,6-di-deoxy-glucopyranose and two units of 2-acetamido-2-deoxy-gulopy-ranuronamide, one of which is acetylated at C-3 position, as shown below:

→4)-α-l-Gul*p*NAc3AcAN-(1→3)-β-d-Qui*p*NAc-(1→4)-α-l-Gul*p*NAcAN-(1→3)-β-d-Qui*p*NAc-(1→ (Molinaro et al. 2003).

### Lipopolysaccharide of *Pseudomonas reactans*

SDS PAGE electrophoresis of water and phenol extracts arising from phenol-water extraction of strain NCPPB1311 of *P. reactans* cells showed that only the phenol phase had the typical banding pattern of smooth LPS. Methylation analysis of the latter fraction revealed the presence of terminal-Glc and 4-substituted-Glc units and, furthermore, its ^1^H NMR spectrum showed an anomeric signal at 5.41 ppm, as broad singlet, in addition to carbinolic signals in the range 3.48–4.21 ppm. All these data suggested an amylose-like structure characterised by an average-molecular weight of 300 KDa as obtained by gel-permeation cromatography.

The presence of 2-keto-3-deoxy-d-mannooctanoic acid (Kdo), dodecanoic acid (12:0), 3-hydroxydecanoic acid [10:0 (3-OH)], 3-hydroxydodecanoic acid, [12:0 (3-OH)] in the phenol phase confirmed the LPS nature of this fraction. The mild-acid hydrolysis of the fraction removed, as precipitate, the lipid A moiety leaving in the supernatant, the saccharide part which, purified by gel-permeation chromatography, gave rise to two fractions; the one in the void volume represented the polysaccharidic part.

**Figure 19:**
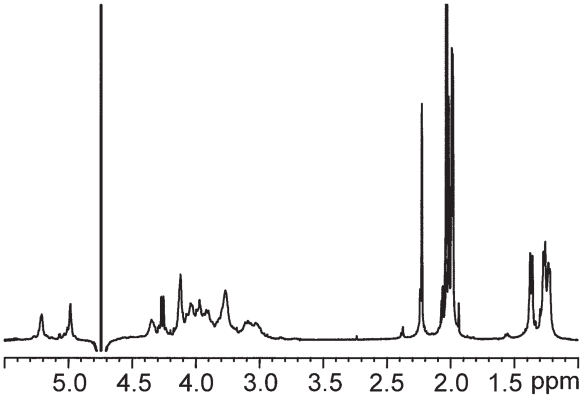
^1^H NMR spectrum of the O-chain polysacharide from *Pseudomonas reactans.*

**Figure 20:**
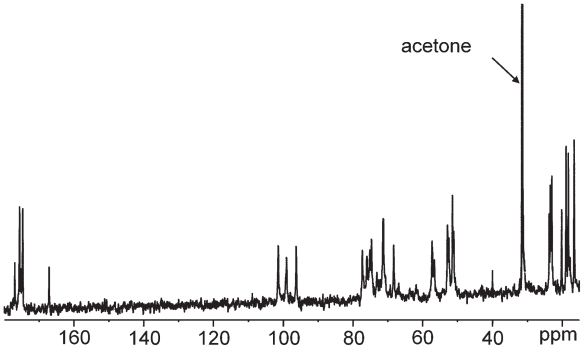
^13^C NMR spectrum of the O-chain polysacharide from *Pseudomonas reactans.*

The GC-MS analysis of the high-molecular-weight polymer showed the presence of Glc*p*NAc, Qui*p*NAc4NAc, and alanine (Ala) and the d configuration of both sugar residues and the l configuration of Ala. The methylation data indicated the presence of 3-substituted glucosamine arising from Glc*p*2Am and the 3-substituted bacil-losamine. The ^1^H and ^13^C NMR spectra (Figs. 19 and 20) showed three anomeric signals of the same integral intensity, suggesting a trisaccharide repeating unit. Sugar moieties are named with **A**–**C** letters in order of decreasing chemical shift (Table 8).

The anomeric configurations, on the basis of their chemical shift values positively confirmed by their ^3^*J*_H-H_ and ^1^*J*_C-H_ values measured from a coupled HSQC experiment, were established to be α, α and β for **A**, **B** and **C** residues, respectively. The complete assignment of all proton and carbon signals was achieved by 2D homonuclear and heteronuclear experiments (DQF-COSY, TOCSY, ROESY, HSQC, HMBC). The ^13^C NMR spectrum was also informative in showing several signals of carbons bearing nitrogen including two signals assigned to a C=N and a CH_3_ group, respectively, of an acetamidino group (Am), the presence of which was established by chemical and spectroscopic work carried out on the O-chain. The localisation of the acetoamidino group was inferred by the ROESY spectrum.

The proton and carbon signals of the two alanine residues (named ala1 and ala2) were clearly identified starting from nitrogen-bearing carbon, which were correlated in the HMBC with the methyl doublets. Moreover, the correlations between the methine proton of each alanine residue with two carbonyl carbons, in turn correlated to methyl acetyl signals, indicated that both the alanine residues were acetylated. The location of both alanine units on the residue C was positively established by the observation of the connectivities observed in the HMBC spectrum. These data were positively confirmed by NOE contacts found in the ROESY spectrum.

The other two acetyl signals present in the ^1^H and ^13^C spectra were located at C-2 and C-4 of the residue A on the basis of the long-range correlation observed in the HMBC spectrum. The sequence of the residues was deduced by ROESY spectrum, whereby each anomeric proton showed an inter-residual contact with the corresponding proton at the glycosylated positions.

These data, together with the downfield shift of the respective carbons and with the expected correlations found in the HMBC spectrum, validated the proposed structure (Table 8). The peculiarity of these monosaccharides, together with other characteristics, will allow further insight into *P. reactans* classification (Molinaro et al. 2002).

### Lipid A of *Pseudomonas reactans*

In animal pathogenic bacteria, lipid A is the endotoxic portion of LPS and its conservative structure usually consists of a glucosamine (GlcN) disaccharide backbone which is phosphorylated at positions 1 and 4’ and is acylated at the positions 2, 3, 2’ and 3’ of the GlcN I (proximal) and GlcN II (distal) residue with 3-hydroxy fatty acids (Zähringer et al. 1999, p 93–114). To date, very little is known about the structure and functions of lipid A in nonanimal associated bacteria (Newmann et al. 2000). Moreover, the study of lipid A structures from nontoxic Gram-negative bacteria is extremely important in order to identify lipid A analogues which can antagonize the biological activation of competent mammalian host cells by lipid A. This was the case of the lipid A of *Rhodobacter capsulatus* and its synthesized analogue labelled as E5531 (Christ et al. 1995).

The LPS extracted from cells of strain NCPPB1311 of *P. reactans* was hydrolysed with AcOH or AcONa to obtain the lipid A moiety. Both conditions gave the same lipid A composition as judged by MALDI-TOF spectrometry and compositional analysis. Compositional analysis further revealed the presence of a phosphate and GlcN. Methylation analysis of the de-phosphorylated and reduced sample showed the presence of 6-substituted GlcN*ol* and terminal GlcN. The absolute configuration of the GlcN was demonstrated to be d.

Fatty acid analysis revealed the presence of (*R*)-3-hydroxydodecanoic [C12:0 (3-OH)] exclusively as amides and (*R*)-3-hydroxydecanoic [C10:0 (3-OH)], (*S*)-2-hydroxydecanoic [C12:0 (2-OH)] and dodecanoic acid (C12:0) linked in ester linkage (molar ratio: GlcN, 2; phosphate, 1.6; fatty acids, 5.2).

The amide-linked fatty acids were identified using MALDI-TOF of de-O-acylated lipid A showing the presence of two C12:0 (3-OH) fatty acids at the 2 and 2’ positions of both GlcN residues and a peak lacking one phosphate. Fourthermore GlcN II unit bearing a C12:0 (3-OH) and a phosphate group was identified. Accordingly, a nonstoichiometric phosphate substitution was present on the GlcN II residue.

**Figure 21:**
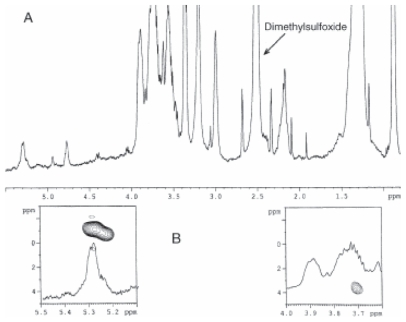
(A) ^1^H NMR spectrum and (B) ^1^H, ^31^P HMQC spectrum of de-O-acylated lipid A from *Pseudomonas reactans*.

The ^1^H NMR spectrum was reported in Figure 21A. A full two-dimensional NMR analysis was performed (COSY, TOCSY, ROESY, HSQC). The NMR data were in agreement with the results obtained by MS. In ROESY spectrum, besides the expected intra-residue correlations typical of the β anomeric configuration, the anomeric proton of GlcN II, showed inter-residue cross peaks with the two protons of GlcN I. These data, together with the downeld shift of the C-6 of GlcN I, proved the β (1→6) linkage between the two sugars. Methylation analysis confirmed the results obtained by NMR.

The phosphate substitution was inferred by a ^1^H, ^31^P HMQC spectrum which indicated the anomeric α-substitution of the GlcN I and the 4’ substitution of the GlcN II (Fig. 21B). Therefore, the de-O-acylated lipid A was demonstrated to be composed of two d-GlcN, two units of fatty acids C12:0 (3-OH) N-linked to both GlcN and phosphate residues at position C-1 and nonstoichiometric at C4. A lipid A aliquot was de-phosphorylated with HF and the product thus obtained was analysed by positive ion MALDI-TOF. Some structural information were obtained on both a hexacyl and pentacyl lipid A and in particular on about the N-linked fatty acid distribution. Similar analyses were carried out on the intact lipid A and ammonium hydroxide treated lipid A fractions. The negative MALDI-TOF spectrum of intact lipid A fraction mainly confirmed its fatty acid heterogeneity.

A Combination of homo- and hetero- two-dimensional NMR experiments (COSY, TOCSY, ROESY, HSQC, HMBC) were performed to assign of the fully acetylated lipid A structure. Starting from the anomeric signals in the TOCSY and COSY spectra it was possible to identify every resonance of each residue.

**Figure 22:**
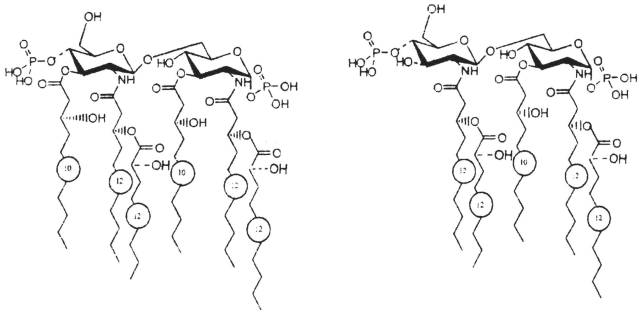
Structure of the main lipid A from *Pseudomonas reactans.*

In conclusion, the main lipid A species (Fig. 22) consisted of a bisphosphorylated GlcN backbone with phosphate groups at C-1 and at C-4’ positions (C-4’ phosphorylation is nonstoichiometric). Fatty acids are linked as amides and esters to C-2, C-3, C-2’ and C-3’, with this last carbon not always substituted. The hexacyl species bears two C12:0 (3-OH) in amide linkage and two C10:0 (3-OH) in ester linkage; the secondary fatty acids, C12:0 (2-OH) or C12:0, are linked to the primary C12:0 (3-OH) amides. The pentacyl species is lacking the C10:0 (3-OH) at position C-3’ of distal glucos-amine (Silipo et al. 2002).

The search for other lipid A structures of Gram-negative bacteria is extremely important in order to obtain possibly non-toxic lipid A molecules which can act as antagonists of lipid A cell response and possibly prevent the septic shock in mammalian cells. To the best of our knowledge this is the first complete lipid A structure elucidated from a mushroom-associated bacterium, and the second from a nonanimal pathogenic organism, after the report on the lipid A structure from *E. carotovora*, a plant-associated Gram-negative bacterium (Fukuoka et al. 1997).

The fatty acid composition of lipid A from *P. reactans* is very close to that of other related *Pseudomonas* species in which the main molecular species harbour five or six fatty acids (Zähringer et al. 1999, p 93–114). The main peculiarity is that in this lipid A the acyl moiety at the C-3’ position of GlcN II is partly missing. Actually, several studies have confirmed the importance of the structure and composition of acyl chains for biological activity and stimulation of mammalian cells. For example *P. aeruginosa* lipid A exhibits a low endotoxic activity mainly because its characteristic fatty acid composition lacks the 3-O-linked fatty acid at GlcN I (Kulshin et al. 1991). In *P. aeruginosa*, *Rhizobium leguminosarum* and *Salmonella typhimurium* a lipase has been found in the external membrane that cleaves this linkage after complete biosynthesis of the lipid A bearing the two Kdo units (Basu et al. 1999; Trent et al. 2001). In analogy, a different lipase should be present in the outer membrane of *P. reactans*, able to cleave selectively the ester bound fatty acid of the distal GlcN. The discovery of this new unidentified enzyme could provide a new biochemical apparatus for selective de-O-acylation and preparation of new lipid A derivatives which can have a different effect on immune stimulation in animal systems.

This chemical peculiarity in bacteria could play an important role in the host infection process. In fact, plants have been found to have systems of innate immunity (Newmann et al. 1997; Newmann et al. 1995), and it is intriguing that, in *R. leguminosarum*, the absence of the 3-O-acyl fatty acid helps the bacterium to evade the host’s response while the plant can still defend itself from other Gram-negative infections (Basu et al. 1999). The absence of 3’-O-acyl fatty acid in the unusual lipid A of *P. reactans* might be a strategy by which the bacterium eludes the immune response. Further studies are needed to confirm this hypothesis (Silipo et al. 2002).

### Lipopolysaccharide of *Burkholderia gladioli* pv. *agaricicola*

A neutral O-specific polysaccharide (OPS) was obtained by mild acid hydrolysis of the lipopoly-saccharide (LPS) of strain ICMP1096 of *B. gladioli* pv. *agaricicola.*

Sugar analysis of the polysaccharide identified rhamnose, mannose and galactose in an approximate molar ratio of 5:2:2. Methylation analysis revealed the polymer to be composed of 2-substituted rhamnopyranose and two 3- and 4-substituted hexopyranose residues.

**Figure 23:**
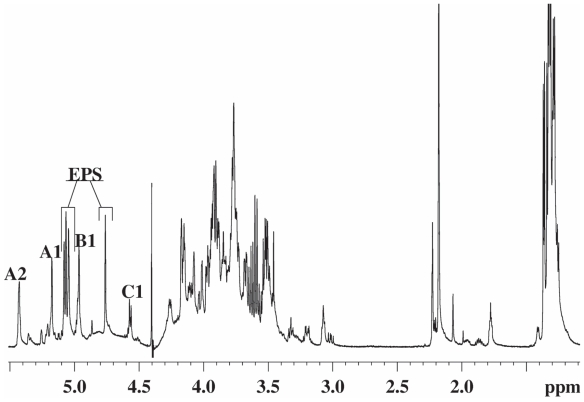
^1^H NMR spectrum of the O-specific polysaccharide OPS of the LPS from *Burkholderia gladioli* pv. *agaricicola*.

The absolute configurations of the sugars were identified as d-mannose, d-galactose and d-rhamnose. The ^1^H NMR spectrum (Fig. 23) of the O-specific polysaccharide contained four main signals in the anomeric region, a signal characteristic for a methyl group of rhamnose and one signal characteristic for an *O*-acetyl group. The sugar residues were labelled **A** to **C** according to decreasing chemical shifts of the anomeric protons. The couplings of the anomeric signals of residues **A** and **B** identified the *manno* and that of residue **C** the *galacto* configured residues. A COSY experiment allowed to locate the acetoxy group at C-2 of the mannose residue.

Data from sugar analysis and from NMR spectroscopy indicated that the OPS consisted of a trisaccharidic repeating unit, containing one residue each of d-mannose, d-rhamnose and d-galactose.

The complete structural characterization of the polysaccharide was achieved by 1D and 2D ^1^H and ^13^C NMR spectroscopy. ^1^H,^1^H COSY, double-quantum-filtered COSY (DQFCOSY), ROESY and TOCSY, as well as ^1^H,^13^C HMQC and HMBC spectra allowed the complete assignment of all ^1^H and ^13^C chemical shifts.

The anomeric configurations of all residues were assigned by the coupling constants ^1^*J*_H-1,C-1_ which were identified in another ^1^H,^13^C HMQC experiment recorded without decoupling. The ^1^*J*_H-1,C-1_ revealed that the mannosyl and rhamnosyl residues had the *α*- and the galactosyl residue the *β*-anomeric configuration.

The ROESY and ^1^H,^13^C HMBC experiments revealed the sequence of the sugar residues in the repeating unit.

In summary, the data identified the structure of the O-specific polysaccharide from *B. gladioli* pv. *agaricicola* as:


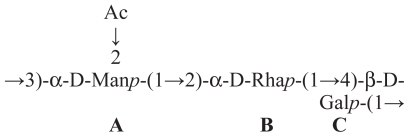


This structure was also confirmed carrying out similar chemical and spectroscopic studies on the deacetylated polysaccharide obtained by alkaline hydrolysis of the OPS (Karapetyan et al. 2006).

In conclusion, the structure of the chemical repeating unit of the OPS of the LPS from *B. gladioli* pv. *agaricicola* was established and it represents a novel repeating unit not found in the OPS of plant-pathogenic bacteria. Rhamnose is a common sugar in such OPS, a number of rhamnan backbones in OPS of LPS from plant pathogenic *Xanthomonas* and *Pseudomonas* species have been identified (Knirel and Kochetkov, 1994; Jansson, 1999, p 155–78; Corsaro et al. 2001; Evidente and Motta, 2002, p 581–629).

### Exopolisaccharides of *Burkholderia gladioli* pv. *agaricicola*

A putative capsular polysaccharide containing d-rhamnose was isolated from cells of strain ICMP1096 of *B. gladioli* pv. *agaricicola.* The structure of the exopolysaccharide was determined by chemical analyses and NMR spectroscopy.

**Figure 24:**
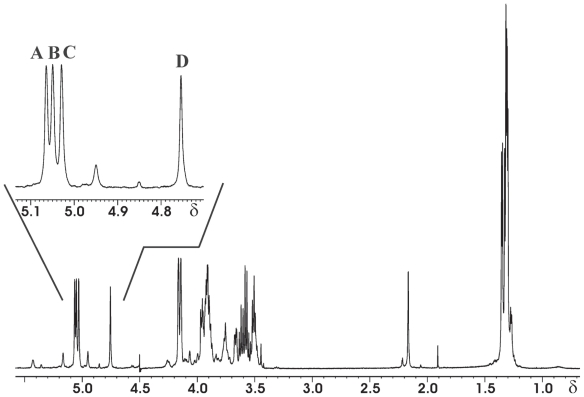
^1^H NMR spectrum of the capsular polysaccharide isolated from *Burkholderia gladioli* pv. *agaricicola*.

Sugar analyses of the polysaccharide identified d-rhamnose as the sole constituent. The ^1^H NMR spectrum (Fig. 24) of the capsular polysaccharide showed four anomeric signals and four overlapping methyl signals characteristic for 6-deoxy-sugars. The sugar residues were labelled **A** to **D** in order to decreasing chemical shifts of the anomeric protons.

The ^13^C NMR spectrum contained 20 signals, however, since four of them possessed double intensity, a total of 24 carbon atoms were present, thus confirming a repeating unit comprising four hexoses. A ^1^H,^13^C-heteronuclear multiple-quantum coherence (HMQC) experiment identified four anomeric carbon signals.

The anomeric configurations of all rhamnose residues were assigned by the coupling constants ^1^*J*_H-1,C-1_ which were identified in another ^1^H,^13^C HMQC experiment without decoupling. The complete structural characterization of the capsular polysaccharide was achieved by 1D and 2D ^1^H and ^13^C NMR spectroscopy. ^1^H,^1^H COSY, double-quantum-filtered COSY (DQFCOSY) and TOCSY, as well as ^1^H,^13^C HMQC spectra allowed the complete assignment of all ^1^H and ^13^C chemical shifts.

The 2D rotating frame nuclear Overhauser spectroscopy (ROESY) and ^1^H,^13^C-heteronuclear multi bond correlation (HMBC) experiments revealed the sequence of the sugar residues in the repeating unit.

In summary, the data identified the structure of a putative capsular polysaccharide from *B. gladioli* pv. *agaricicola* as (Kaczynsky et al. 2006):


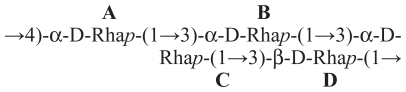


## Figures and Tables

**Table 1:** Antimicrobial activity of tolaasin I and WLIP towards bacteria.

Bacteria	No strains	Minimal Inhibitory Quantity (μg)	
		Tolaasin I	WLIP
**Gram positive**			
*Bacillus megaterium*	1	0.04	0.32
*Rhodococcus fascians*	2	0.16	1.28
*Clavibacter michiganensis* subsp. *michiganensis*	2	0.32	0.64
*C. michiganensis* subsp. *sepedonicus*	1	0.32	0.64
*Curtobacterium flaccunfaciens*	1	0.16	0.64
**Gram negative**			
*Escherichia coli*	1	0.64	-b
*Pseudomonas tolaasii*	1	-a	“
*P.* “*reactans*”	1	1.28	“
*P. syringae* pv. *phaseolicola*	1	1.28	“
*P. corrugata*	1	0.64	“
*Agrobacterium tumefaciens*	1	0.64	“
*Erwinia herbicola*	1	0.64	“
*Xanthomonas campestris* pv. *phaseoli*	1	1.28	“

a no inhibitory activity were observed with a quantity of tolaasin I equal at 1.28 μg.

b no inhibitory activity were observed with a quantity of WLIP equal at 10.24 μg.

**Table 2:** Antimicrobial activity of tolaasin I and WLIP towards phytopathogenic fungi, chromista, yeast and filamentous fungi responsible for mycoses of mammalian, and cultivated mushrooms.

	No strains	Minimal Inhibitory Quantity (μg)	
		Tolaasin I	WLIP
*Fusarium solani*	3	0.32	5.12
*F. oxysporum*	1	0.32	2.56
*F. graminearum*	1	0.16	-a
*Sclerotinia sclerotiorum*	2	0.32	1.28
*S. minor*	1	0.16	0.64
*Sclerotium rolfsii*	1	0.32	-a
*Botrytis cinerea*	2	0.16	1.28
*Rhizoctonia solani*	1	0.08	0.64
*Verticillium dahliae*	1	0.16	1.28
*Trichoderma viride*	1	0.16	1.28
*Phytopthora nicotianae*	1	0.32	-a
*P. citrophthora*	1	0.32	-a
*Armillaria mellea*	2	0.32	2.56
*Heterobasidion annosum*	2	0.16	1.28
*Rhodotorula pilimanae*	1	0.64	-a
*Candida albicans*	1	5.12	-b
*C. parapsilosis*	1	-a	“
*Malassezia pachydermatis*	1	-a	“
*Cryptococcus neoformans*	1	2.56	“
*Agaricus bisporus*	2	0.08	1.28
*Pleurotus eryngii*	3	0.32	2.56
*P. ostreatus*	2	0.16	1.28
*Lentinus edodes*	2	0.32	1.28

a no inhibitory activity were observed with a quantity of tolaasin I or WLIP equal to 10.24 μg.

b no inhibitory activity were observed with a quantity of WLIP equal at 25 μg.

**Table 3:** Minimal haemolytic quantity (M.H.Q.) of tolaasin I or WLIP as determined in agarose plate assay at different temperatures.

Lipodepsipeptide quantity (μg)	Average Diameter of the Haemolysis Area (mm)			
	Tolaasin I		WLIP	
	25 °C	37 °C	25 °C	37 °C
10.24	8	16	24	24
5.12	7	11	22	22
2.56	7	7	18	18
1.28	0	0	11	11
0.64	0	0	9	9
0.32	0	0	0	0

**Table 4:** Antimicrobial activity of tolaasins I, II, and A–E.

Micro-organism	Tolaasins Minimal Inhibitory Quantity (μg)						
	I	II	A	B	C	D	E
*Rizoctonia solani* 1583	0.32	0.64	1.28	2.56	5.12	0.16	5.12
*Rhodotorula pilimanae* ATCC26423	2.56	5.12	5.12	>5.12	> 5.12	2.56	>5.12
*Bacillus megaterium* ITM100	0.32	0.64	1.28	2.56	>5.12	0.16	2.56
*Rodococcus fascians* NCPPB3067	0.32	0.64	1.28	1.28	> 5.12	0.16	2.56
*Escherichia coli* K12 ITM103	>5.12	>5.12	>5.12	>5.12	>5.12	>5.12	> 5.12
*Erwinia carotovora* subsp. *carotovora* ICMP5702	>5.12	>5.12	>5.12	>5.12	>5.12	>5.12	> 5.12

**Table 5:** Permeabilizing activity of WLIP and tolaasin I on vesicles of different lipid composition and a statistical analysis of their pore-formation mechanism.

Lipid Composition^a^ (mol%)			WLIP				Tolaasin I			
PC	SM	sterol	1/C_50_^b^(μM^−1^)	K_1_^c^ (M^−1^)	M^d^	K_2_^e^	1/C_50_^b^ (μM^−1^)	K_1_^c^ (M^−1^)	M^d^	K_2_^e^
100	0	0	0.59 ± 0.04	50 ± 20	8 ± 1	533 ± 451	2.8 ± 0.4	233 ± 52	6 ± 1	5.2 ± 2.6
50	50	0	1.41 ± 0.05	100 ± 30	6 ± 1	200 ± 340	4.9 ± 0.2	673 ± 179	6 ± 1	1.7 ± 1.1
50	33	16.5*	0.70 ± 0.05	100 ± 30	12 ± 1	217 ± 247	2.8 ± 0.2	200 ± 50	6 ± 1	12 ± 12
50	16.5	33*	0.59 ± 0.01	50 ± 20	8 ± 1	533 ± 451	1.2 ± 0.2	100 ± 30	7 ± 1	19.7 ± 17.4
50	0	50*	0.45 ± 0.01	50 ± 20	11 ± 1	533 ± 451	0.4 ± 0.1	50 ± 20	7 ± 1	0.8 ± 0.3
50	33	16.5^§^	0.70 ± 0.03	100 ± 30	12 ± 1	413 ± 440	2.5 ± 0.2	250 ± 54	7 ± 1	2.8 ± 1.7
50	16.5	33^§^	0.59 ± 0.08	50 ± 20	8 ± 1	533 ± 451	1.3 ± 0.1	100 ± 30	7 ± 1	30 ± 20
50	0	50^§^	0.54 ± 0.01	50 ± 20	10 ± 1	533 ± 451	1.0 ± 0.2	50 ± 20	5 ± 1	30 ± 20

a Lipid mixtures are reported on a molar base. PC: egg phosphatidylcholine; SM: sphyngomyelin; the sterol included in the vesicles was cholesterol* and ergosterol^§^.

b The permeabilising activity is expressed as the reciprocal of the concentration of toxin causing 50% of calcein release (C_50_). Data are average ± S.D. of at least two different experiments.

c The partition coefficient of toxin monomers into the liposome.

d The number of monomers necessary for the formation of an active pore.

e The aggregation process of membrane-inserted monomers.

c; d ; e Data are average ± S.D. of at least three different experiments.

**Table 6:** FTIR spectroscopy determination of the secondary structure of tolaasin I in aqueous buffer, with and without lipids, and in different solvents.

	% Secondary structure^a^		
System	β	h	r
buffer	29	44	27
TFE	24	45	30
HFIP	24	48	28
SDS	27	47	26
POPC	21	63	15

a β: total β-structure (i.e. β-sheet, β-turn); h: helix; r: random. Errors of FTIR determinations are ±5%.

**Table 7:** Assignment and dichroic ratio of some IR bands observed in lipid vesicles with and without toxin.

Wavenumber (cm^−1^)	Vibration^a^	Direction (*θ*)^b^	Dichroic Ratio	Angle (γ_⊥_)^c^
*Lipid alone*				
2920	as CH_2_ stretching	90°	1.32 ± 0.01	33° ± 1°
2850	s CH_2_ stretching	90°	1.34 ± 0.01	34° ± 1°
*LUVs + WLIP*				
2920	as CH_2_ stretching	90°	1.48 ± 0.01	41° ± 1°
2850	s CH_2_ stretching	90°	1.52 ± 0.01	42° ± 1°
*LUVs + tolaasin I*				
2920	as CH_2_ stretching	90°	1.61 ± 0.01	45° ± 1°
2850	s CH_2_ stretching	90°	1.63 ± 0.01	45° ± 1°
1660	Amide I’ helix	30°	2.51 ± 0.10	47° ± 2°

a as: asymmetric; s: symmetric.

b Direction of the variation of the dipole moment associated to the vibration with respect to the direction of the main molecular axis (aliphatic chain or α-helix axis).

c Average angle between the direction of the molecular axis and the perpendicular to the crystal plane (i.e. membrane plane).

**Table 8:** **^1^**H and **^13^**C NMR resonance for each residue and substituent of the O-chain from *Pseudomonas reactans.*

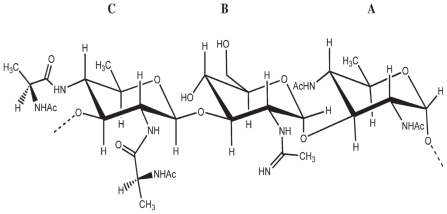						
Residue	H-1/C-1	H-2/C-2	H-3/C-3	H-4/C-4	H-5/C-5	H-6/C-6
Qui*p*NAc4NAc	5.199	4.039	3.933	3.751	3.893	1.239
(**A**)	95.8	52.1	74.4	56.4	67.4	16.4
Acetyl (×2)		1.987				
	174.0	23.2				
GlcAm	4.971	4.103	3.777	3.969	3.986	3.866, 3.854
(**B**)	98.0	50.4	76.6	74.6	72.5	61.1
Amidino group		2.209				
	166.6	19.7				
Qui*p*NAlaAc4NAlaAc	4.325	3.745	3.766	3.579	3.461	1.191
(**C**)	100.7	56.8	76.5	56.1	71.0	17.6
Ala1 residue		4.239	1.324			
	176.6	51.0	18.0			
Ala2 residue		4.099	1.274			
	177.4	51.9	17.0			
Ac Ala1		1.983				
	174.2	22.7				
Ac Ala2		1.973				
	176.3	22.8				
